# Kar5p Is Required for Multiple Functions in Both Inner and Outer Nuclear Envelope Fusion in *Saccharomyces cerevisiae*

**DOI:** 10.1534/g3.114.015800

**Published:** 2014-12-02

**Authors:** Jason V. Rogers, Mark D. Rose

**Affiliations:** Department of Molecular Biology, Princeton University, Princeton, New Jersey 08544-1014

**Keywords:** yeast mating, karyogamy, Brambleberry, Gex1, Tht1

## Abstract

During mating in the budding yeast *Saccharomyces cerevisiae*, two haploid nuclei fuse via two sequential membrane fusion steps. SNAREs (*i.e.*, soluble N-ethylmaleimide–sensitive factor attachment protein receptors) and Prm3p mediate outer nuclear membrane fusion, but the inner membrane fusogen remains unknown. Kar5p is a highly conserved transmembrane protein that localizes adjacent to the spindle pole body (SPB), mediates nuclear envelope fusion, and recruits Prm3p adjacent to the SPB. To separate Kar5p’s functions, we tested localization, Prm3p recruitment, and nuclear fusion efficiency in various *kar5* mutants. All domains and the conserved cysteine residues were essential for nuclear fusion. Several *kar5* mutant proteins localized properly but did not mediate Prm3p recruitment; other *kar5* mutant proteins localized and recruited Prm3p but were nevertheless defective for nuclear fusion, demonstrating additional functions beyond Prm3p recruitment. We identified one Kar5p domain required for SPB localization, which is dependent on the half-bridge protein Mps3p. Electron microscopy revealed a *kar5* mutant that arrests with expanded nuclear envelope bridges, suggesting that Kar5p is required after outer nuclear envelope fusion. Finally, a split-GFP assay demonstrated that Kar5p localizes to both the inner and outer nuclear envelope. These insights suggest a mechanism by which Kar5p mediates inner nuclear membrane fusion.

During mating in the budding yeast *Saccharomyces cerevisiae* two haploid cells of opposite mating type (a and α) fuse to form a diploid. Mating requires the sequential processes of shmooing (polarized growth toward the mating partner), adhesion, cell wall degradation, cell fusion, nuclear congression, and nuclear fusion (karyogamy; reviewed in Ydenberg and Rose 2008; Merlini *et al.* 2013). Nuclei congress along microtubules that emanate from the half-bridge adjacent to the spindle pole body (SPB) of each nucleus (congression; reviewed in Gibeaux and Knop 2013). The nuclear envelope comprises an outer nuclear membrane (ONM) that is continuous with the endoplasmic reticulum (ER), a lumenal space, and an inner nuclear membrane (INM). Nuclear fusion requires the sequential fusion of both the ONM and INM; these steps are not simultaneous (Melloy *et al.* 2007).

Kar5p in *Saccharomyces cerevisiae* is an integral membrane protein that primarily resides in the lumen of the nuclear envelope (NE) (Beh *et al.* 1997). Kar5p is essential for nuclear fusion, although its specific role has remained unclear. Initial electron microscopy suggested that *kar5-486* mutant zygotes were blocked after the initiation of ONM fusion but before dilation of a membrane bridge connecting the two nuclei (Beh *et al.* 1997). Later electron tomography revealed that *kar5-486* mutant zygotes are blocked equally often before and after ONM fusion (Melloy *et al.* 2009). Moreover, the appearance of the nuclear envelope in the *kar5-486* mutant zygotes suggested that Kar5p also might function in coupling the INM and ONM near the SPB (Melloy *et al.* 2009). Taken together, these observations suggested that Kar5p might have roles in multiple processes during nuclear fusion. Recently, functional Kar5p orthologs have been identified in zebrafish (*Danio rerio*), green alga (*Chlamydomonas*), and the malaria parasite (*Plasmodium*), suggesting that Kar5p is a member of a deeply conserved protein family with members in almost all eukaryotic clades (Abrams *et al.* 2012; Ning *et al.* 2013).

Genetic screens have identified at least 10 other genes with mutations that disrupt nuclear fusion: *SEC20*, *UFE1*, *USE1*, *BOS1*, *PRM3*, *KAR8*, *KAR2*, *SEC66*, *SEC72*, and *SEC63*. The first four proteins are ER-localized soluble N-ethylmaleimide–sensitive factor attachment protein receptors (SNAREs) that mediate efficient nuclear fusion, putatively between the ONMs (Rogers *et al.* 2013). Prm3p is a small (133-residue) peripheral membrane protein that localizes exclusively to the nuclear envelope and mediates ONM fusion (Melloy *et al.* 2009; Shen *et al.* 2009). Kar8p/Jem1p is a nonessential ER-lumenal DnaJ-like chaperone required for INM fusion (Kurihara *et al.* 1994; Nishikawa and Endo 1997; Melloy *et al.* 2009). Kar2p/BiP is an essential, highly conserved ER-lumenal ATPase that mediates protein import into the ER and acts as an chaperone for protein folding in the ER (Normington *et al.* 1989; Rose *et al.* 1989; Vogel *et al.* 1990; Sanders *et al.* 1992; Simons *et al.* 1995). *kar2-1* mutants are blocked at INM fusion under conditions permissive for normal growth rate, suggesting that its role in nuclear fusion is somewhat different from its essential mitotic role (Melloy *et al.* 2009). Kar8p and Kar2p likely act together at the same step during nuclear fusion, as Kar8p overexpression can suppress a *kar2-1* nuclear fusion defect (Brizzio *et al.* 1999). Sec66p, Sec72p, and Sec63p, along with Kar2p, form a translocation complex in the ER (Brodsky and Schekman 1993; Panzner *et al.* 1995; Young *et al.* 2001) and have moderate nuclear fusion defects (Ng and Walter 1996). However, Kar5p is not expressed at normal levels in a *sec66* mutant, suggesting that the translocation mutants do not mediate nuclear fusion directly but instead mediate Kar5p insertion and stability (Brizzio *et al.* 1999).

Importantly, the only three proteins known to affect INM fusion are Kar5p, Kar8p, and Kar2p. However, Kar8p and Kar2p are both NE/ER-lumenal and not bound to either nuclear membrane, they are thus unlikely to mediate INM fusion directly. Therefore, Kar5p is the most likely candidate to mediate INM fusion. Here we demonstrate that Kar5p has at least three functions: recruitment of Prm3p to the SPB, coupling of the ONM and INMs, and a third function, acting after ONM fusion, that is required for INM fusion. We additionally demonstrate that Kar5p localization near the SPB requires the half-bridge component Mps3p and that the accumulation of Kar5p requires Prm3p. Consistent with roles in the coupling and fusion of both the INM and ONM, Kar5p localizes to both faces of the nuclear envelope near the SPB.

## Materials and Methods

### Strains and general yeast methods

**Figure 1:**
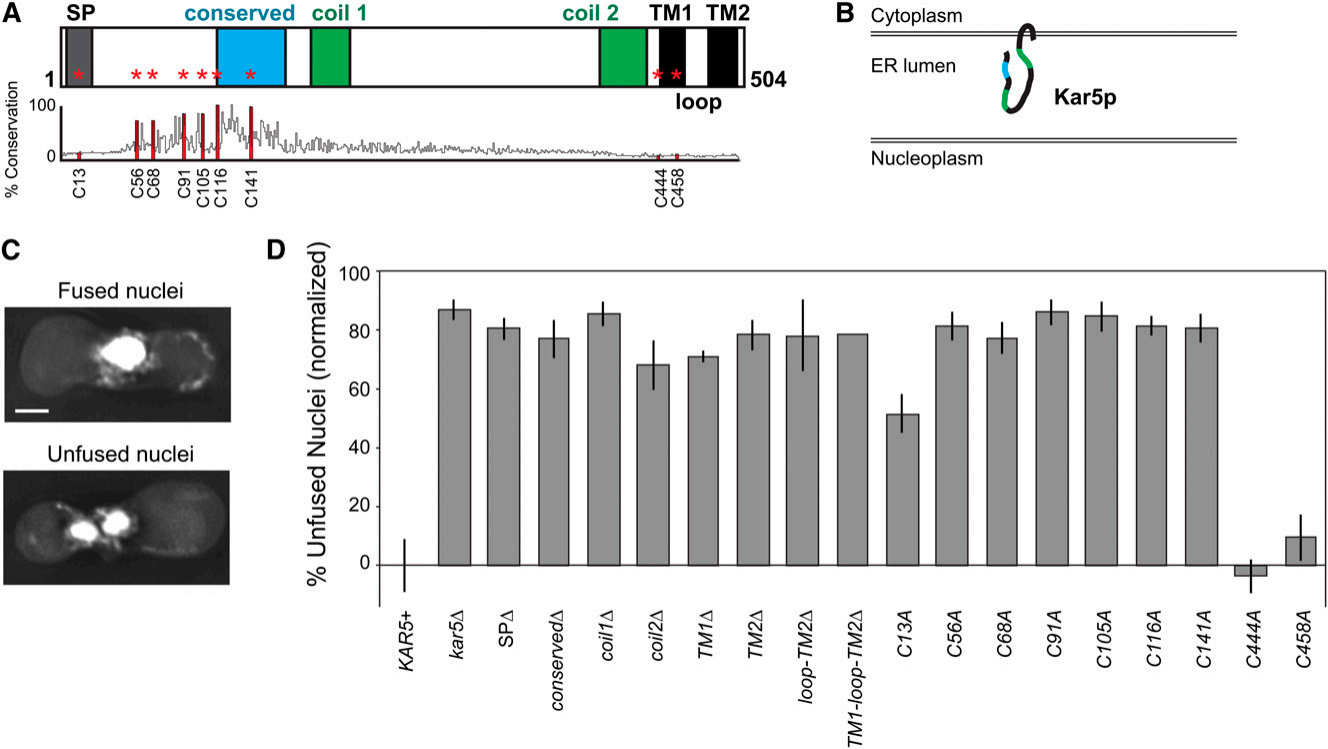
All major Kar5p domains are essential for nuclear fusion. (A) Schematic of Kar5p predicted domains (Beh *et al.* 1997). Cysteines are marked with a red asterisk. Percent conservation is the percent identity to the consensus sequence among 96 Pfam sequences (Family Tht1, PF04163). SP refers to signal peptide (4−23). Coil1 and coil2 refer to two predicted coiled-coil domains (186−215 and 401−436). The conserved domain runs from residues 116 to 167. TM refers to transmembrane domains (TM1 445−465, TM2 481−504). The loop domain refers to the small cytoplasmic loop between the TM domains (466−480). Specific residues deleted in each construct are listed in the strain table (Table S1). (B) Schematic of Kar5p predicted topology based on protease protection assays and computational predictions (Beh *et al.* 1997). Note that SP is likely cleaved and thus not shown. The lumenal secondary structure is completely speculative. (C) Representative examples of fused and unfused nuclei. Scale bar, 2 μm. (D) Nuclear fusion assay. All crosses were *kar5*Δ (MS7670) × *kar5*Δ (MS7673). MS7670 contained a CEN plasmid with the indicated *kar5* alleles. To remove the effects of plasmid loss, values were normalized against KAR5+ (29% unfused nuclei). Average of multiple independent experiments are shown (at least three trials for each, except two trials for SPΔ, conservedΔ, coil2Δ, TM1Δ, TM1-loop-TM2Δ, and C458A). Error bars show ± SEM.

All strains and plasmids are listed in Supporting Information, Table S1; strains beginning with MS are isogenic to S288C. Deletion endpoints are listed in the legend to Figure 1 and in Table S1. Standard methods, including cell culture and transformations, were used as described in Amberg *et al.* (2005). Most plasmid cloning was performed directly in yeast via homologous recombination (Oldenburg *et al.* 1997), except *kar5* deletion and point mutation plasmids, which were created *in vitro* using polymerase chain reaction−mediated, site-directed mutagenesis (Hemsley *et al.*1989).

### Nuclear fusion assays

Nuclear fusion assays were performed as described in Gammie and Rose (2002). To summarize, cells were grown to log phase and 0.5 OD units of each strain were mixed on a 0.45-μm nitrocellulose filter (EMD Millipore, Billerica, MA) and incubated on a YEPD plate at 30° for 2.75 hr. Cells were then washed from the filter and fixed in 3:1 methanol:acetic acid and stained with 1 μg/mL DAPI.

### Image acquisition and processing

All fluorescence microscopy was performed on a DeltaVision deconvolution microscope (Applied Precision, Issaquah, WA), based on a Nikon TE200 (Melville, NY) with an inverted 100X NA 1.4 objective, a 50-W mercury lamp, and a Photometrics Cool Snap HQ CCD camera (Photometrics, Tucson, AZ). Image pixel size is 49.2 × 49.2 nm. All images were deconvolved using the Applied Precision SoftWoRx imaging software.

For nuclear fusion assay microscopy (fixed, DAPI-stained cells), we acquired large z-stacks that captured the entire cell (typically 19 slices separated by 0.2 μm). The remaining imaging experiments used live cells in growth medium with smaller z-stacks (typically ~9 slices separated by 0.2 μm) to avoid photobleaching.

### Quantitative image analysis

Quantitative image analysis was performed using custom ImageJ macros. In summary, cells were manually outlined, and then fluorescence measurements within each cell were performed on background-subtracted, summed-intensity projections. To define percent green fluorescent protein (GFP) present at the SPB, the SPC42-mCherry (SPB marker) punctum was automatically outlined, and then the outlined region was expanded uniformly with a radius of 0.25 μm. The amount of GFP in this larger area was defined as “at the SPB.”

### Electron microscopy

Transmission electron microscopy on serial sections was performed as described in Gammie and Rose (2002). In summary, we mated cells as described for nuclear fusion assays and then fixed in 2% glutaraldehyde for 30 min at room temperature. Downstream processing included 4% potassium permanganate staining for 4 hr at 4°, sodium periodate treatment, uranyl acetate staining, and embedding in LR-white resin. Ultrathin serial sections (~80 nm) were created using a Leica UC6 ultramicrotome. Sections were placed on a nickel-slotted grid (Formvar film, FF-2010-Ni, Electron Microscopy Sciences, Hatfield, PA) and imaged directly, without lead citrate staining. Imaging was performed on a Zeiss LEO Omega 912 EF-TEM.

## Results

### All major Kar5p domains are essential for nuclear fusion

Previous studies on Kar5p identified two predicted coiled-coil domains and three hydrophobic domains (Beh *et al.* 1997), of which the first is likely to be a cleaved signal peptide necessary for insertion into the ER (SignalP-4.1 prediction; Petersen *et al.* 2011). In addition, a “conserved domain” with homology to the *S. pombe tht1* gene resides in the N-terminal region (Tange *et al.* 1998). Protease protection experiments demonstrated that the majority of Kar5p is ER-lumenal, with two transmembrane domains and a cytoplasmic loop near the C-terminus (Beh *et al.* 1997; Figure 1, A and B). We compared the sequence conservation between Kar5p and 95 orthologs (Pfam Family Tht1, PF04163) and noted that the residues with the greatest conservation were a series of six cysteines within a more broadly conserved region near the N-terminus (Figure 1A). This region is synonymous with the recently defined cysteine-rich domain conserved in evolutionarily distant Kar5p orthologs in protists, plants, and vertebrates (Ning *et al.* 2013).

To determine which Kar5p domains are essential for its function, we assayed nuclear fusion by mating cells and counting the number of zygotes containing unfused nuclei (Figure 1C). We assayed *kar5* mutants lacking single domains or with cysteine-to-alanine mutations. All of the predicted domains and the conserved cysteines were essential for Kar5p function (Figure 1D). Two of the nonconserved cysteines, C444 and C458, had no effect on nuclear fusion when mutated to alanine, and the third nonconserved cysteine, C13, had an intermediate effect. The appearance of the unfused nuclei (shape, internuclear distance, etc.) did not differ between the mutants (data not shown).

### Kar5p recruits Prm3p to the nuclear fusion zone and has additional functions

**Figure 2:**
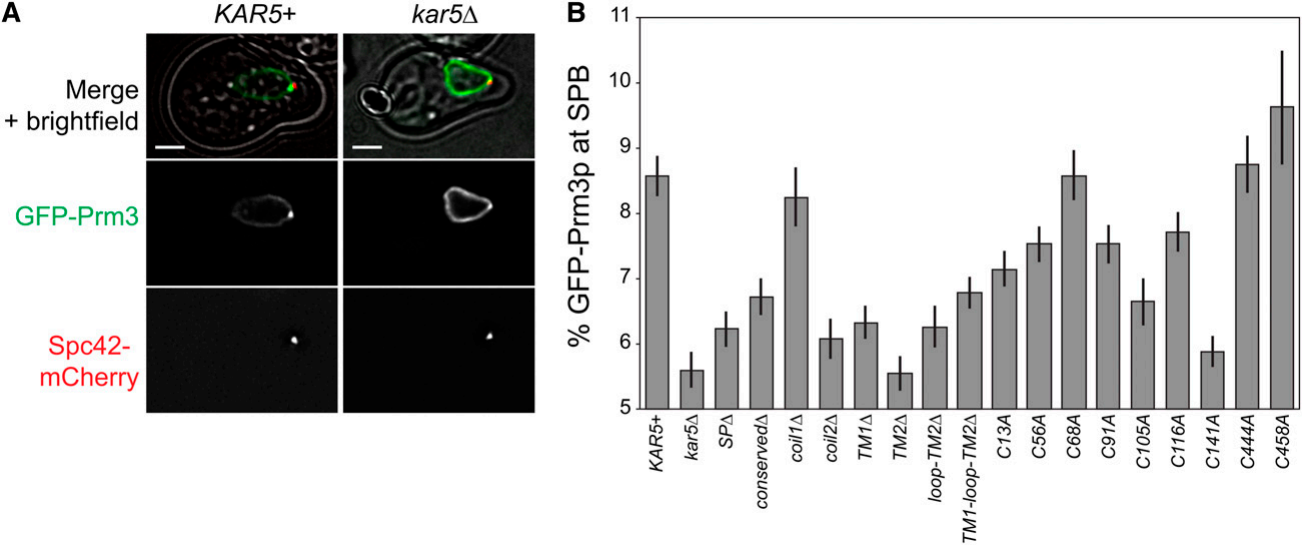
Prm3p enrichment assay in *kar5* mutants. (A) Representative example of GFP-Prm3p recruitment to the SPB (marked by Spc42-mCherry). The merged panels include a transmitted light image. GFP images are displayed at the same brightness. Scale bar, 2 μm. (B) Percent of total GFP-Prm3p present near the SPB (see the section *Materials and Methods* for details on quantification) for the indicated genotypes in alpha-factor−treated cells (~2 hr, 30°). Strains used are MS8389 plus the indicated CEN plasmids. Average and ± SEM are shown. Data are pooled from two independent experiments. GFP, green fluorescent protein.

Although all Kar5p domains were essential for nuclear fusion, we reasoned that Kar5p might perform multiple functions within nuclear fusion, and therefore each domain may have different specific functions. Previous studies demonstrated that Prm3p localization near the SPB is partially Kar5p-dependent and that Prm3p interacts with Kar5p through the Kar5p transmembrane and/or cytoplasmic loop domains (Shen *et al.* 2009; Figure 2A). We therefore measured GFP-Prm3 localization near the SPB (marked with Spc42-mCherry) in *kar5* mutants (Figure 2B). The amount of GFP-Prm3 was largely unaffected in the various *kar5* mutants (see Figure S1 for full dataset comparing SPB enrichment to total GFP-Prm3 expression). As expected, GFP-Prm3p was significantly more enriched near the SPB in *KAR5*+ cells than in *kar5*Δ (8.6 ± 0.3% for *KAR5*+, 5.6 ± 0.3% for *kar5*Δ, *P* < 0.001 by *t*-test). In *kar5*Δ, Prm3p was consequently more diffusely localized over the nuclear envelope. The *C444A* and *C458A kar5* mutants, which had no nuclear fusion defects, exhibited normal GFP-Prm3p recruitment. Consistent with previous studies (Shen *et al.*, 2009), our data confirmed the requirement of the Kar5p C-terminal transmembrane domains for Prm3p recruitment. The *kar5-coil2*Δ and *C141A* mutants also were strongly defective for Prm3p recruitment, and most of the remaining mutants exhibited intermediate phenotypes. Interestingly, the *kar5-coil1*Δ and *C68A* mutants recruited Prm3p as well as wild-type, despite being unable to mediate nuclear fusion; this demonstrates that Kar5p must have additional functions beyond recruiting Prm3p, consistent with a potential additional role for Kar5p in INM fusion.

### Kar5p-conserved region is required for its SPB localization

**Figure 3:**
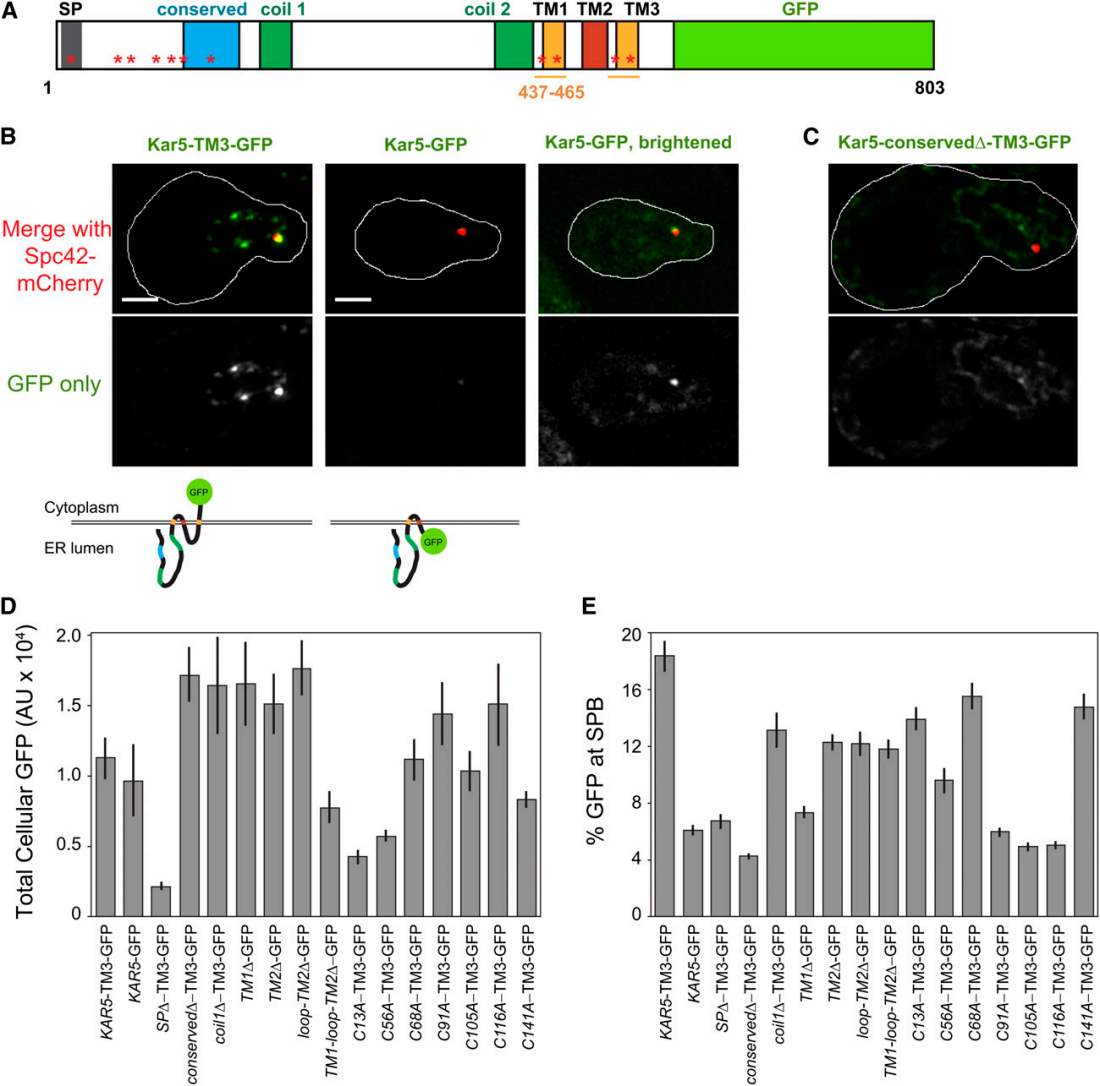
Mutant Kar5p localization. (A) Schematic of Kar5-TM3-GFP. The region between coil2 and the end of TM1 (residues 437-465) were duplicated to create TM3. The region between TM3 and GFP contains a FLAG tag and a glycine-serine linker. (B) Representative examples of Kar5-TM3-GFP and Kar5-GFP along with schematics of their membrane topology (bottom). The first two panels (left and middle) are displayed at the same pixel intensities. The right panel is brightened to show the GFP localization. Cell outlines are drawn on the top panel. Scale bars, 2 μm. (C) Example of the lack of SPB-localization observed in Kar5-conserved Δ-TM3-GFP. Image is displayed at the same image brightness as the left panel in B. (D) Quantification performed as in Figure 2B, except total cellular GFP is shown. Strains used are MS8020 plus the indicated CEN plasmid. Average and ± SEM are shown. Data are pooled from two independent experiments. (E) As in D, but the percent of total GFP near the SPB is shown. GFP, green fluorescent protein; SPB, spindle pole body.

Nuclear fusion occurs adjacent to the SPB, where Kar5p and Prm3p are enriched, presumably at the half-bridge (Melloy *et al.* 2007). We next asked whether any of the Kar5p domains or conserved cysteines are required for Kar5p’s stability or localization near the SPB. We initially created a simple Kar5-linker-GFP fusion, but noted that it was dim and its localization did not match previous Kar5p immunofluorescence experiments (Beh *et al.* 1997). We reasoned that this might be due to an inability of the GFP to fold in the ER lumen, or the GFP might disrupt necessary interactions between the Kar5p C-terminus and another protein. To resolve this issue, we noted that several homologs of Kar5p have a fourth hydrophobic domain (*e.g.*, Tht1p from *S. pombe*) that would lead to the C-terminus being located in the cytoplasm. Accordingly, we added another transmembrane domain (referred to as TM3) at the C-terminus of Kar5p, by duplicating the second hydrophobic domain prior to adding the GFP, allowing the GFP to fold outside of the ER-lumen (cytoplasm or nucleoplasm, depending on Kar5p orientation; Figure 3A). The Kar5-TM3-GFP largely rescued nuclear fusion function (Figure S2A), and appeared brighter than the version with GFP in the ER-lumen, localized near the SPB, and formed additional puncta along the nuclear envelope (Figure 3B), consistent with Kar5p immunofluorescence (Beh *et al.* 1997). To verify that the added TM3 domain was acting as a true transmembrane domain, and that the GFP was folding outside of the lumen, we used a His4C-based membrane topology assay (Sengstag 2000). In this assay, the His4C histidinol dehydrogenase enzyme (converts histidinol to histidine) allows *his4−* cells to grow on medium lacking histidine but containing histidinol. When fused to an ER membrane protein, such as Kar5p, the His4C enzyme must face the cytoplasm or nucleoplasm to confer growth. Consistent with our topology predictions, Kar5-TM2Δ-His4C and Kar5-TM3-His4C supported growth on histidinol, whereas Kar5-His4C did not (Figure S2B).

We assayed the localization of the *kar5* mutants after adding TM3-GFP as necessary (the transmembrane domain deletion mutants naturally caused the GFP to reside outside of the lumen and did not require any transmembrane additions). Many *kar5* mutants visually appeared similar to wild-type (bright SPB enrichment, nuclear envelope localization; see Figure S3 for representative images of each mutant). In contrast, Kar5-SPΔ-TM3-GFP appeared extremely dim, supporting the hypothesis that the signal peptide (SP) domain is a signal peptide required for normal Kar5p insertion into the ER and stability. Conversely, Kar5-conservedΔ-TM3-GFP, *C91A*, *C105A*, and *C116A* expressed well and localized to the nuclear envelope but did not enrich at the SPB (Figure 3, C and E). This finding suggests that the Kar5p-conserved domain and possibly the preceding cysteines mediate an interaction that anchors Kar5p near the SPB. To gain quantitative insight into intrinsic stability and SPB enrichment for each *kar5* mutant, we quantified total cellular GFP intensity and percent GFP adjacent to the SPB. We confirmed that most mutants expressed as well as wild-type Kar5-TM3-GFP, except *kar5-SP*Δ, which had the weakest level of expression, and the *C13A* and *C56A* mutants, which had intermediate levels (Figure 3D). We measured protein levels in a subset of mutants by α-GFP western blot and obtained results consistent with the quantitative microscopy (Figure S4). Because the *C13A* mutation is within the SP, it is not surprising that it has an intermediate expression defect, and, consequently, an intermediate nuclear fusion defect (Figure 1D). Quantification of SPB enrichment, which inherently normalizes for different levels of total GFP, confirmed a strong enrichment defect in the *conserved*Δ, *C91A*, *C105A*, and *C116A* mutants, and revealed an intermediate defect in the *TM1*Δ and *C56A* mutants (Figure 3E). A comparison of total GFP and SPB enrichment for each mutant is shown in Figure S5. These data reveal distinct domains of Kar5p responsible for expression/stability and SPB localization.

### Prm3p induces Kar5p aggregation along the nuclear envelope

**Figure 4:**
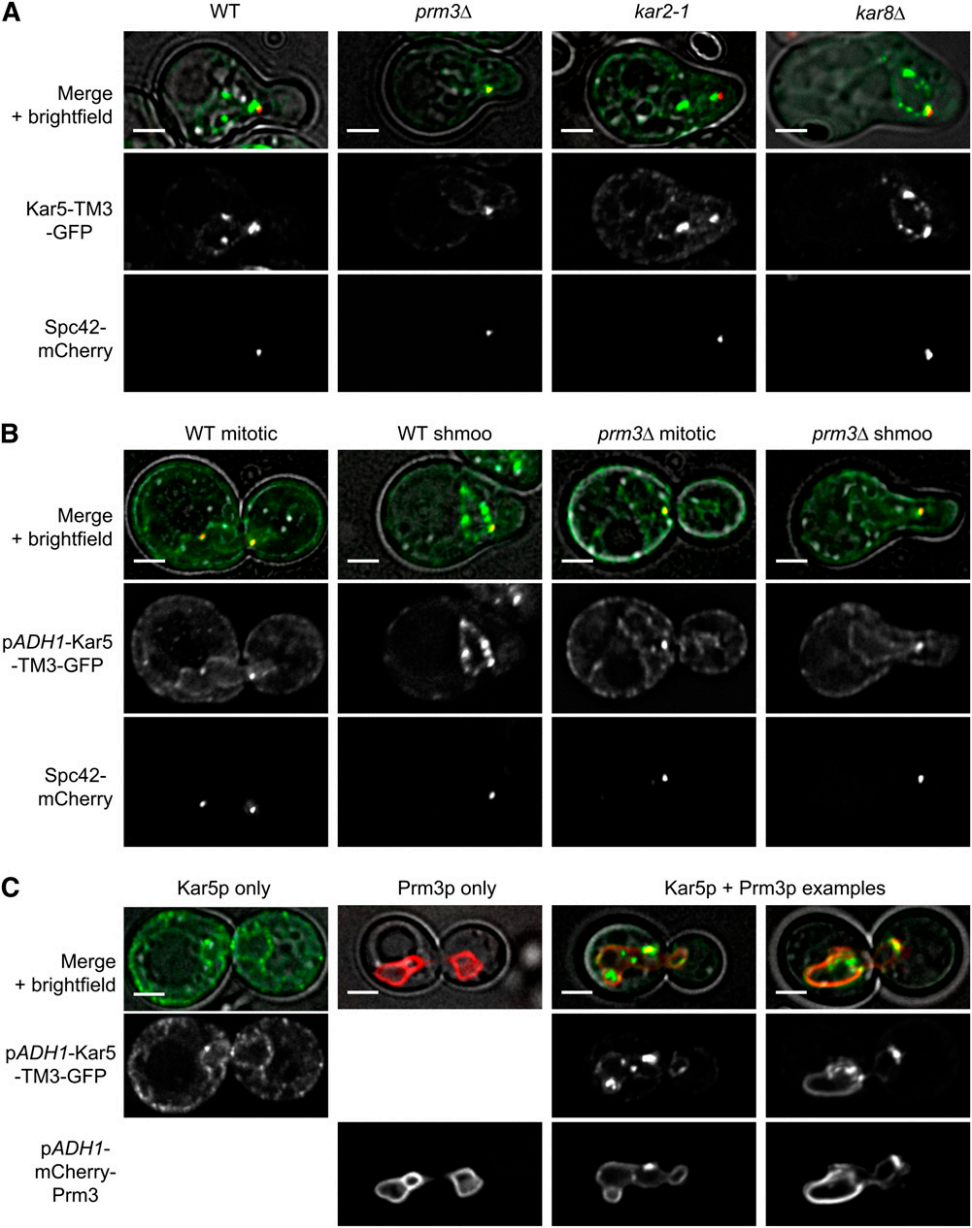
Prm3p induces Kar5p aggregation along the nuclear envelope. (A) Representative examples of Kar5-TM3-GFP (pMR6364) in wild type (WT; MS7729), *prm3*Δ (MS7884), kar2-1 (MS8041), and *kar8*Δ (MS8087) shmoos. (B) Representative examples of pADH1-Kar5-TM3-GFP (pMR6371) in WT and *prm3*Δ mitotic cells and shmoos. (C) Representative examples of pADH1-Kar5-TM3-GFP and pADH1-mCherry-Prm3 (pMR6433) in MS7670, either separately or coexpressed. GFP images within each panel are displayed at the same brightness. Scale bars, 2 μm. GFP, green fluorescent protein.

To further understand how Kar5p might interact with other nuclear fusion proteins, we expressed Kar5-TM3-GFP in nuclear fusion mutants *prm3*Δ, *kar2-1*, and *kar8*Δ. In *kar2-1* and *kar8*Δ shmoos, Kar5-TM3-GFP appeared normal; it enriched strongly at the SPB, and formed several bright puncta elsewhere on the nuclear envelope (Figure 4A). In contrast, in *prm3*Δ shmoos Kar5-TM3-GFP enriched strongly at the SPB, but did not form additional puncta, and instead was diffusely localized along the nuclear envelope and peripheral ER (Figure 4A). This suggested that Prm3p might have a role in restricting Kar5p to the nuclear envelope and causing it to aggregate.

To further understand what factors contribute to Kar5p localization, we expressed Kar5-TM3-GFP under the control of the constitutive *ADH1* promoter. Because Kar5p is normally only induced by pheromone, using the *ADH1* promoter allowed us to visualize Kar5p in mitotic cells that are not expressing additional pheromone-induced proteins. We observed that Kar5-TM3-GFP still enriched at the SPB in mitotic cells, demonstrating that the SPB-enriching factor is not restricted to pheromone treated cells (Figure 4B). Furthermore, Kar5-TM3-GFP was diffusely localized along the nuclear envelope and peripheral ER, rather than aggregated in puncta along the nuclear envelope (Figure 4B), as expected if Prm3p, which is also pheromone-induced, is required for Kar5p aggregation. Consistent with this, we observed that adding pheromone to *PRM3*+ cells constitutively expressing Kar5-TM3-GFP induced puncta formation along the nuclear envelope in shmoos, whereas in *prm3*Δ shmoos the Kar5-TM3-GFP distribution remained unchanged (Figure 4B). Therefore, Prm3p is necessary for Kar5p aggregation and restriction to the nuclear envelope. To determine whether Prm3p is sufficient for Kar5p aggregation in the absence of other pheromone-induced genes, we used the *ADH1* promoter to simultaneously express both Kar5-TM3-GFP and mCherry-Prm3p in mitotic cells. When coexpressed, we observed a Kar5p localization pattern similar to that seen in shmoos and observed frequent coaggregation of both Kar5p and Prm3p, demonstrating that Prm3p is sufficient to induce Kar5p aggregation along the nuclear envelope (Figure 4C).

### Mps3p is required for Kar5p localization near the SPB and efficient nuclear fusion

**Figure 5:**
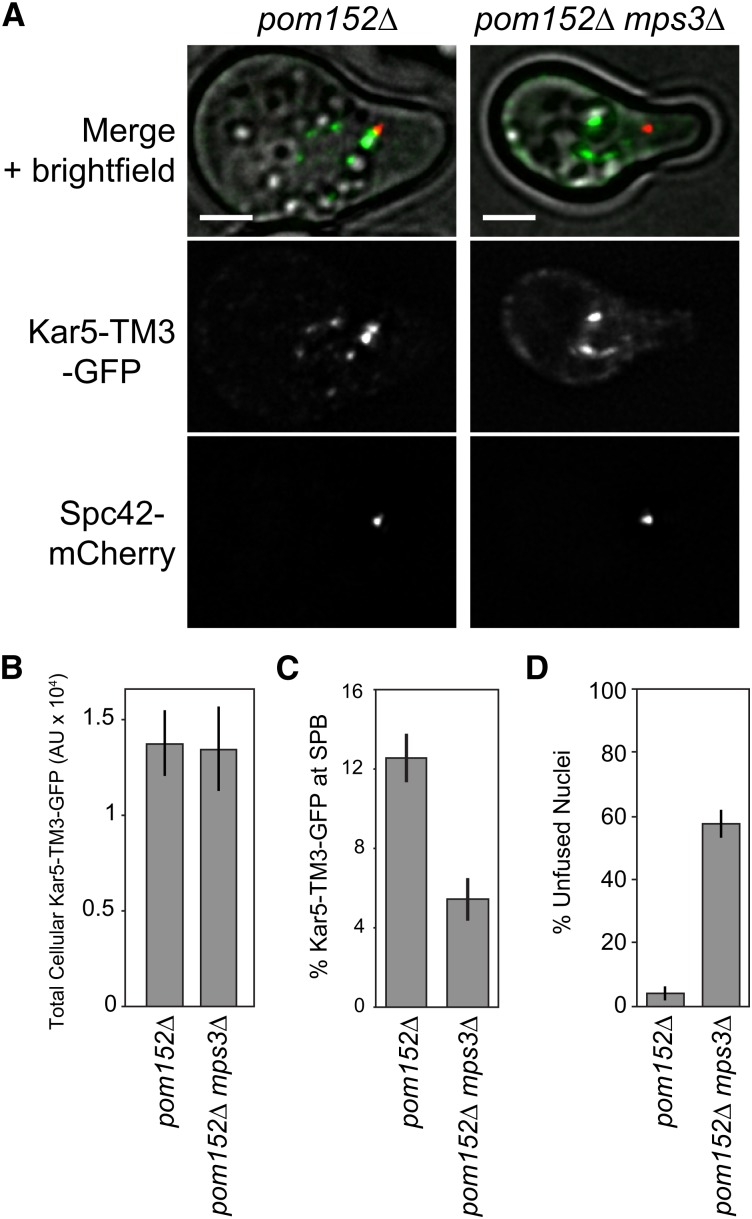
Mps3p is required for Kar5p localization to the SPB and efficient nuclear fusion. (A) Representative examples of Kar5-TM3-GFP in *pom152*Δ (MY14635) and *pom152*Δ *mps3*Δ (MY14637). Note the lack of GFP enrichment near the SPB marker. GFP images are displayed at the same brightness. Scale bars, 2 μm. (B) Quantification of total cellular GFP as in Figure 3D. Average and ± SEM are shown for *pom152*Δ (n = 36) and *pom152*Δ *mps3*Δ (n = 12). (C) As in B, but percent GFP near the SPB is shown. (D) Nuclear fusion assay as in Figure 1D. Crosses are *pom152*Δ (MY14348) x *pom152*Δ (MY14349) and *pom152*Δ *mps3*Δ (MY14355) × *pom152*Δ *mps3*Δ (MY14357). Data are pooled from two independent experiments; average and binomial standard error are shown. GFP, green fluorescent protein; SPB, spindle pole body.

Because Kar5p localizes near the SPB, and because the SPB-enriching factor is also present in mitotic cells, we suspected that Kar5p is anchored by an SPB half-bridge protein. Only four proteins are known to comprise the half-bridge: Kar1p, Cdc31p, Sfi1p, and Mps3p (reviewed in Jaspersen and Winey 2004). Of these, only Mps3p has an extended NE-lumenal domain. Moreover, Mps3p interacts with Kar8p, an ER-lumenal chaperone required for nuclear fusion, and *mps3-7* mutants have a mild nuclear fusion defect (37% zygotes contain apposed but unfused nuclei; Nishikawa *et al.* 2003). We therefore suspected that Mps3p might mediate Kar5p-anchoring. Although *mps3*Δ cells are inviable, *pom152*Δ *mps3*Δ double mutants are viable and grow as well as wild-type (Witkin *et al.* 2010) Therefore, we examined Kar5-TM3-GFP localization and found that it was unaffected in *pom152*Δ single mutants but it completely lacked SPB-enrichment in *pom152*Δ *mps3*Δ (Figure 5A). Quantitatively, total cellular Kar5-TM3-GFP was unaffected, but percent GFP near the SPB decreased from 12.5 ± 1.2% to 5.4 ± 1.1% (Figure 5, B and C). We speculated that if Kar5p localization is disrupted in *pom152*Δ *mps3*Δ, then these mutants should also have a strong nuclear fusion defect. Indeed, 58% (n = 118 zygotes, SD 5%) of *pom152*Δ *mps3*Δ zygotes had unfused nuclei, compared with 4% (n = 74 zygotes, SD 2%) of *pom152*Δ zygotes (Figure 5D). Nuclear congression was not affected in the *pom152*Δ *mps3*Δ zygotes (average internuclear distance 0.4 μm, compared to 0.3 μm in *kar5*Δ zygotes and 2.3 μm in congression-defective *kar1*-Δ*15* zygotes; *kar5*Δ and *kar1*-Δ*15* data from Rogers *et al.* 2013). These data suggest that Mps3p plays a key role in nuclear envelope fusion by anchoring Kar5p near the SPB, where the two nuclei initially meet, although it remains possible that Mps3p has additional roles in nuclear fusion that are Kar5p-independent.

### *kar5-C68A* zygotes are blocked at INM fusion

**Figure 6:**
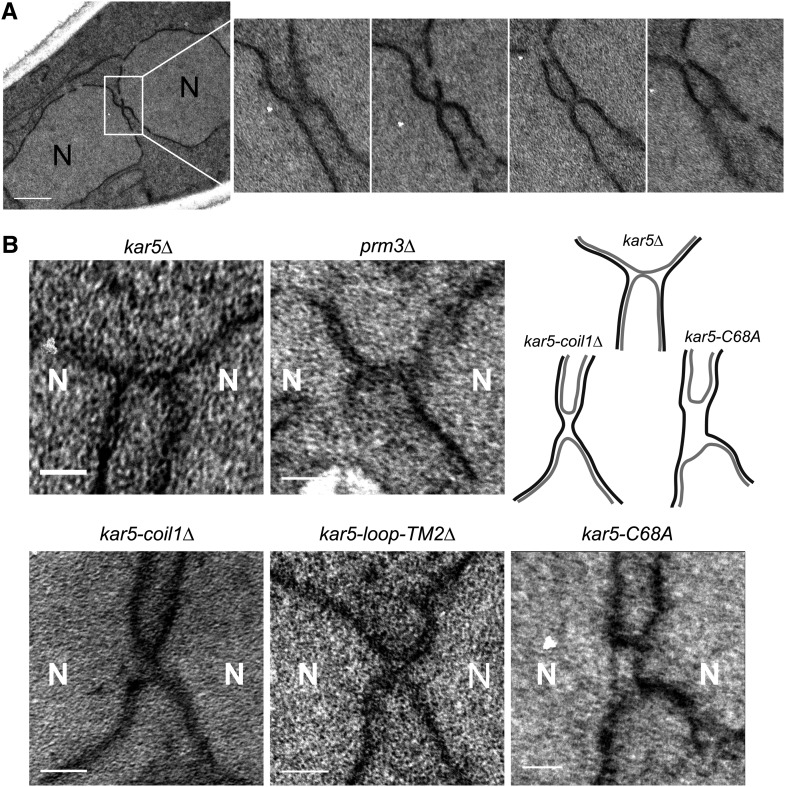
Electron microscopy of kar5 mutants that may mediate distinct functions. (A) Four representative serial sections (~80 nm thick) of a *kar5-loop-TM2*Δ zygote (see the section *Materials and Methods* for details). Note the tight membrane bridge observed in the second serial section. N = nucleus. Scale bar, 500 nm. (B) Representative membrane bridges from other *kar5* mutants. Each zygote pair is *kar5*Δ (MS7670) × *kar5*Δ (MS7673) with the indicated CEN plasmid in both parents. *kar5*Δ indicates that the strain is carrying an empty CEN vector (pMR1868). For comparison with a mutant arrested at outer membrane fusion, we also imaged *prm3*Δ (MS7590) × *prm3*Δ (MS7591). An interpretive cartoon of the *kar5*Δ, *kar5-coil1*Δ and *kar5-C68*A is shown. Outer nuclear envelopes are in gray, inner nuclear envelopes are in black. All zygotes are oriented with the two nuclei along the horizontal axis. Scale bars, 100 nm.

Because *kar5 coil1*Δ and *C68A* mutants appeared completely normal by assays of expression, SPB enrichment, and Prm3p recruitment but were still defective for nuclear fusion, we speculated that these mutants might be affected in a more direct, catalytic role in nuclear fusion. To gain insight into their nuclear fusion defects, we performed serial-section electron microscopy (Figure 6A) on *kar5*Δ, *prm3*Δ, *kar5-coil1*Δ, *kar5-loop-TM2*Δ, and *kar5-C68A* zygotes (Figure 6B). The *kar5*Δ zygotes frequently contained a membrane bridge between the nuclei that had not expanded (of 19 zygotes, seven contained unambiguous membrane bridges, two did not; the remaining 10 were ambiguous due to glancing sections). The *prm3*Δ and *kar5-loop-TM2*Δ mutants appeared similar to *kar5*Δ. In some of the *kar5-coil1*Δ zygotes, the INMs appeared to be closely abutting over a wide region (average membrane bridge width 59 ± 7 nm, n = 6), as opposed to the narrower but long bridges observed for *kar5*Δ zygotes (average width 35 ± 5 nm, n = 7; *P* = 0.03 compared with *kar5-coil1*Δ by 2-sided *t*-test). In contrast, the *kar5-C68A* mutant zygotes consistently contained a wide and rectangular membrane bridge with a clear lumen (of seven unambiguous bridges, six appeared wide and rectangular; Figure S6). This phenotype suggests that the *kar5-C68A* zygotes completed ONM fusion but were blocked before INM fusion. Importantly, because these zygotes are consistently blocked after ONM fusion, Kar5p must mediate later steps required for INM fusion.

### Kar5p localizes to both nuclear envelope faces

**Figure 7:**
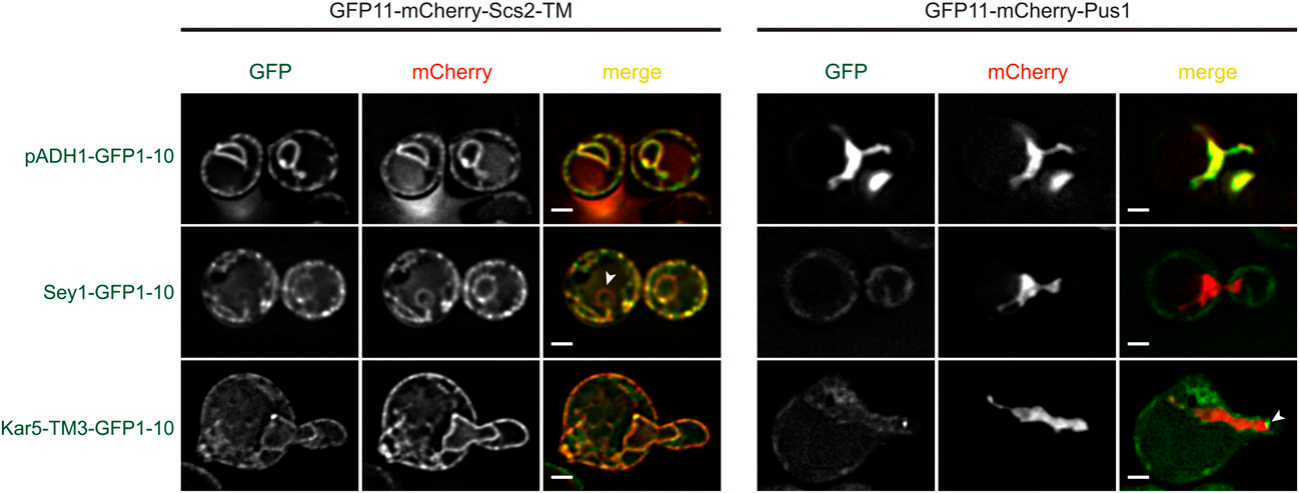
Kar5p localizes to both nuclear envelope faces. Indicated CEN GFP1-10 plasmids were expressed in strain MY14667 (GFP11-mCherry-Pus1) or MY14668 (GFP11-mCherry-SCS2-TM). Green fluorescence indicates that GFP1-10 and GFP11 can colocalize and consequently fluoresce. Note that Sey1-GFP1-10 only fluoresces with the Scs2-TM marker and is excluded from the nucleus (arrowhead), demonstrating outer/inner nuclear envelope specificity in this assay. In Kar5-TM3-GFP1-10, note the bright fluorescence (arrowhead) near the shmoo tip when combined with GFP11-mCherry-Pus1. Because different fluorescent reporters were used (with differing expression levels), images are not displayed at the same brightness. Scale bars, 2 μm.

Because Kar5p seems to mediate INM fusion, and might couple together the INMs and ONMs, we suspected that Kar5p must reside in the INM. However, because Kar5p interacts with Prm3p, a protein that resides on the ONM, Kar5p must also reside on the ONM. To test the possibility that Kar5p resides on both the INM and ONMs, we used a split-GFP−based assay that determines the orientation of nuclear envelope proteins (S. Jaspersen, personal communication). In this assay, the GFP11 fragment is fused to Scs2-TM, which resides in the ONM and ER, or Pus1p, which localizes exclusively to the nucleoplasm and can interact exclusively with INM-bound proteins. The protein of interest is tagged with the GFP1-10 fragment, and its localization is determined by the observation of fluorescence with either GFP11-Scs2-TM, which indicates that the query protein resides on the ONM, or GFP11-Pus1, which indicates that the query protein resides in the INM. As a control, we observed that a constitutively expressed GFP1-10 fragment (p*ADH1*-GFP1-10) fluoresced uniformly with both GFP11-containing proteins (Figure 7). When GFP1-10 was fused to Sey1p, a protein known to associate predominantly with the peripheral ER network (Hu *et al.* 2009), Sey1-GFP1-10 fluoresced specifically with GFP11-Scs2-TM throughout the ER and was excluded from the nucleus, as expected, and did not fluoresce with GFP11-Pus1. When Kar5-TM3-GFP1-10 was coexpressed with GFP11-Scs2-TM, we observed bright puncta and diffuse staining along the nuclear envelope, consistent with its normal localization. Weaker uniform fluorescence was also observed along the cell periphery, suggesting that Kar5p can diffuse into the peripheral ER. When combined with GFP11-Pus1, Kar5-TM3-GFP1-10 fluoresced brightly at a single punctum near the shmoo tip, suggesting that Kar5-TM3-GFP1-10 is also present on the INM, localized near the SPB. These results demonstrate that Kar5p resides on both nuclear envelope faces, and suggest that almost all inner membrane-associated Kar5p resides near the SPB.

## Discussion

Here we demonstrated that Kar5p has multiple roles during nuclear fusion, including Prm3p recruitment, nuclear membrane coupling, and at least one other function required for INM fusion. Reciprocally, Prm3p promotes Kar5p accumulation in the nuclear envelope. Kar5p localization to the SPB is Mps3p-dependent, and requires the Kar5p conserved domain. A summary of our results and proposed functions is shown in Table 1. These observations suggest a model for the assembly of Kar5p at the SPB and indicate a role for Kar5p at multiple stages in nuclear envelope fusion.

**Table 1:** Summary of Kar5p experiments

	GFP Expression	SPB Enrichment	Prm3p Recruitment	Fusion	Function
KAR5	+	++	+	+	
SP	−	+/−	−	−	Stability
Conserved	++	−	+/−	−	SPB interaction
Coil1	+	+	+	−	Fusion?
Coil2	ND	ND	−	−	?
TM1	+	+/−	−	+/−	Prm3p recruitment
TM2	+	+	−	−	
Loop-TM2	++	+	−	−	
TM1-loop-TM2	+	+	+/−	−	
C13A	+/−	+	+/−	+/−	Stability
C56A	+/−	+/−	+/−	−	?
C68A	+	+	+	−	Envelope link
C91A	+	−	+/−	−	SPB interaction?
C105	+	−	+/−	−	SPB interaction?
C116A	+	−	+	−	SPB interaction?
C141A	+	+	−	−	Prm3p recruitment?
C444A	ND	ND	+	+	
C458A	ND	ND	+	+	

Phenotype levels from highest to lowest are ++, +, +/−, and −. ND, no data. Raw *P*-values associated with this table are shown in Table S2. GFP, green fluorescent protein; SPB, spindle pole body.

### A model for Kar5p at the SPB

**Figure 8:**
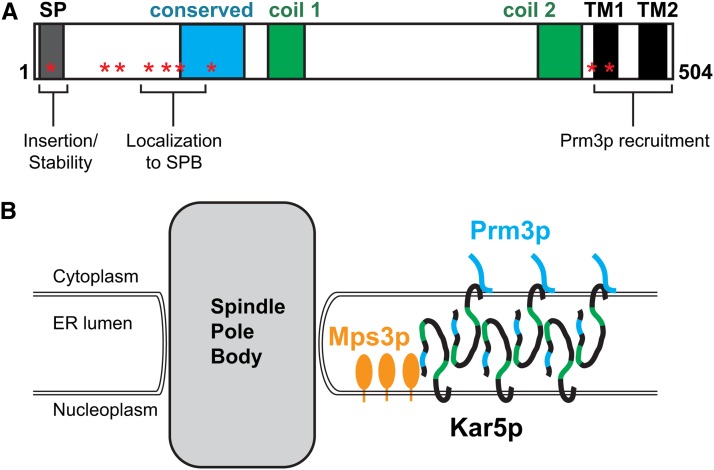
Summary and model for Kar5p localization and functions. (A) Schematic summarizing results of Kar5p experiments, as in Table 1. (B) In this model, Mps3p anchors Kar5p on the inner nuclear membrane near the SPB at the half-bridge, where nuclear fusion typically occurs, and this acts as a seed that initiates further Kar5p aggregation nearby. Cytoplasmic-facing Kar5p proteins recruit Prm3p. Kar5p-Kar5p interactions serve to couple the two nuclear envelope faces together.

We propose that Mps3p anchors Kar5p (directly or indirectly) on the INM at the half-bridge, and this acts as a seed that initiates further Kar5p oligomerization (Figure 8). Oligomerization is supported by the observation that Kar5p, detected by immunofluorescence and GFP, forms extended patches rather than distributing diffusely along the nuclear envelope. Using Kar5-TM3-GFP, these patches are far brighter than single fluorophores and must contain many copies of Kar5p. It is likely that lateral oligomerization in the ONM is facilitated by association with Prm3p, as formation of the non-SPB patches is absent in the *prm3*Δ mutant and is induced in mitotic cells by ectopic Prm3p expression. Indeed, because these patches are Prm3p-dependent, we consider it unlikely that they result from a Kar5p interaction with another protein complex (*i.e.*, nuclear pores, chromatin, etc.) or represent karmellae (patches of excessive ER membrane surrounding the nucleus). Finally, because Kar5p appears to couple the two membranes near the SPB and is present in both membranes at the SPB, we propose that Kar5p proteins on opposite faces of the nuclear envelope dimerize or oligomerize *in trans* to link the ONM and INMs. Because we see separation of the membranes in the *C68A* mutant, an attractive possibility is that disulfide bonds between the conserved cysteine residues mediate the Kar5p−Kar5p interactions.

### A mechanism for INM fusion

How Kar5p might mediate INM fusion remains unknown. Previous work implicated Kar5p in membrane bridge dilation after ONM fusion but before INM fusion (Beh *et al.* 1997; Melloy *et al.* 2009). The *kar5-C68A* mutant is blocked with wide bridges, suggesting that this mutation must block downstream of dilation (Figure 6B and Figure S6). However, the two INMs do not become closely apposed after ONM fusion in *kar5-C68A* zygotes, as they do in *kar2-1* and *kar8*Δ mutants (Melloy *et al.* 2009). Possibly, if the ONM and INM do not remain tightly coupled together, the INMs will remain too far apart to fuse, leading to the observed *kar5-C68A* nuclear fusion defect. Alternatively, it is possible that the *kar5-C68A* zygotes are blocked at INM fusion, and the two inner membranes separate as a secondary consequence.

For *kar5-coil1*Δ the ONMs appear to have fused over a wide area, but unlike *kar5-C68A* we cannot detect a lumenal compartment, as the two INMs are too close together. We interpret this mutant as being defective for INM fusion but not nuclear membrane coupling. Because this region of the protein is predicted to form a coiled-coil structure, it is tempting to speculate that this part of Kar5p acts in a SNARE-like mechanism to fuse the two apposed inner membranes. Of course, it remains possible that Kar5p’s primary role in INM fusion is to form a “fusosome” that localizes other proteins and couples the ONM and INMs together, whereas an unknown protein acts as the actual INM fusogen. However, no other candidate protein has been identified.

Previous work demonstrated that Kar5p recruits Prm3p near the SPB and this could account for the *kar5*Δ nuclear fusion defect (Shen *et al.* 2009). Importantly, our work here demonstrated that Kar5p has additional functions beyond Prm3p recruitment. Unexpectedly, however, even the Kar5p mutants that were expressed but did not enrich near the SPB (*i.e.*, conservedΔ, C91A, C105A, and C116A; see Table 1) still increased Prm3p enrichment more than *kar5*Δ, *kar5-SP*Δ and the C-terminal TMΔ mutants. Therefore, it appears that uniformly diffuse Kar5p along the nuclear envelope can still modestly promote Prm3p recruitment near the SPB. The significance of this is unclear, although we note that there is still some Prm3p enrichment at the SPB even in the absence of Kar5p (Figure 3A), suggesting that Prm3p recruitment near the SPB involves both Kar5p-dependent and Kar5p-independent interactions.

Prm3p is necessary and sufficient to induce Kar5p aggregation along the nuclear envelope at sites away from the SPB. However, because nuclear fusion normally occurs adjacent to the SPB (Byers and Goetsch 1975; Melloy *et al.* 2007), the importance of these extra Kar5p aggregates during normal mating is unclear. Notably, Kar5p also contributes to Prm3p enrichment at the SPB, suggesting that their mutual interaction ensures sufficient protein concentrations near the SPB for fusion. Consistent with a role for Prm3p in increasing the local Kar5p concentration, Kar5p overexpression can partially suppress a *prm3*Δ nuclear fusion defect (Shen *et al.* 2009). This observation is understandable if part of the role of Prm3p in nuclear fusion is to modulate Kar5p localization or stability.

The role of Mps3p in nuclear fusion is less clear. The intermediate nuclear fusion defect of the *pom152*Δ *mps3*Δ mutants is consistent with Mps3p only playing a role in recruiting Kar5p or other nuclear fusion proteins near the SPB. However, it remains possible that Mps3p has additional downstream functions, such as SPB fusion.

The *kar2-1* and *kar8*Δ mutants also are blocked at INM fusion; both mutant zygotes contain wide, expanded membrane bridges similar to but more extreme than those observed in the *kar5-C68A* mutants (Melloy *et al.* 2009). However, because Kar2p and Kar8p are not transmembrane proteins it is unlikely that they directly mediate membrane fusion. Instead, an attractive hypothesis is that Kar2p and Kar8p mediate Kar5p’s coupling of the membranes, possibly by promoting disulfide bond formation. They may also mediate the final catalytic step in INM fusion by promoting Kar5p interactions.

An unresolved question is what regulates the fusion of the INMs. Given that Kar5p is present in both the INM and ONMs, one might expect that it could prematurely catalyze the fusion of the INM and ONMs and lead to fenestrations in the envelope. Similarly, mitotic expression might lead to aberrant nuclear membrane fusion events. Because, mitotic Kar5p expression is not toxic, we favor the hypothesis that outer membrane fusion, perhaps mediated by a pheromone-induced protein such as Prm3p, primes Kar5p for INM fusion.

### Conservation of Kar5p functions

Recent work has demonstrated that Kar5p orthologs form a deeply conserved protein family with members in almost all eukaryotic clades, including plants, protists, alga, fungi, and animals (Abrams *et al.* 2012; Ning *et al.* 2013). The orthologs share similar domain architectures, consisting of an N-terminal signal peptide, one or two coiled-coil domains, two or three C-terminal transmembrane domains, and a cysteine-rich domain near the N-terminus. The cysteine-rich domain is the only region of Kar5p that is highly conserved by amino acid sequence; all six cysteines in this domain (C56 through C141 in *S. cerevisiae*) are conserved in every ortholog.

Because of the deep conservation of the domain architecture and cysteines, it is likely that the fundamental Kar5p membrane fusion mechanism is conserved. Indeed, although animals degrade and reform the nuclear envelope during mitosis and zygote formation (and thus do not directly fuse nuclei), many early-stage embryos form karyomeres, where the nuclear envelope reforms around isolated chromosomes. The karyomeres then fuse to reform a single nucleus. Recent work in zebrafish demonstrated that karyomere fusion requires Brambleberry, a Kar5p ortholog (Abrams *et al.* 2012). Interestingly, Brambleberry localizes as prominent puncta at the karyomere-karyomere fusion interface (Abrams *et al.* 2012), similar to our observations in yeast. Although a Kar5p ortholog has not yet been identified in mammals, the general process of nuclear envelope reformation after mitosis likely uses a mechanism similar to yeast nuclear fusion, even if the proteins are not conserved.

In contrast, Prm3p is not well-conserved, even within fungi, suggesting that the role of Prm3p in nuclear fusion is not universally required, or that different proteins, or even Kar5p, can substitute for Prm3p’s function in organisms lacking a Prm3p ortholog. Alternatively, it is possible that Prm3p has orthologs whose sequence is not well conserved outside of fungi, as only the ∼50 C-terminal residues of Prm3p are required for function (Shen *et al.* 2009).

Given Kar5p’s deep conservation, solving the Kar5p-mediated membrane fusion mechanism should prove generally useful for understanding similar membrane fusion events in other eukaryotic organisms.

## Supplementary Material

Supporting Information

## References

[bib1] Abrams E. W. Zhang H. Marlow F. L. Kapp L. Lu S., 2012 Dynamic assembly of brambleberry mediates nuclear envelope fusion during early. Dev. Cell 150: 521–532. 10.1016/j.cell.2012.05.048 PMC3700733 22863006

[bib2] Amberg D. Burke J. Strathern J., 2005 Methods in Yeast Genetics. Cold Spring Harbor Laboratory Press, Cold Spring Harbor, NY.

[bib3] Beh C. T. Brizzio V. Rose M. D., 1997 KAR5 encodes a novel pheromone-inducible protein required for homotypic nuclear fusion. J. Cell Biol. 139: 1063–1076. 9382856 10.1083/jcb.139.5.1063 PMC2140214

[bib4] Brizzio V. Khalfan W. Huddler D. Beh C. T. Andersen S. S., 1999 Genetic interactions between KAR7/SEC71, KAR8/JEM1, KAR5, and KAR2 during nuclear fusion in *Saccharomyces cerevisiae*. Mol. Biol. Cell 10: 609–626. 10069807 10.1091/mbc.10.3.609 PMC25191

[bib5] Brodsky J. L. Schekman R., 1993 A Sec63p-BiP complex from yeast is required for protein translocation in a reconstituted proteoliposome. J. Cell Biol. 123: 1355–1363. 8253836 10.1083/jcb.123.6.1355 PMC2290880

[bib6] Byers B. Goetsch L., 1975 Behavior of spindles and spindle plaques in the cell cycle and conjugation of *Saccharomyces cerevisiae*. J. Bacteriol. 124: 511–523. 1100612 10.1128/jb.124.1.511-523.1975 PMC235921

[bib7] Gammie A. E. Rose M. D., 2002 Assays of cell and nuclear fusion. Methods Enzymol. 351: 477–498. 12073365 10.1016/s0076-6879(02)51866-8

[bib8] Gibeaux R. Knop M., 2013 When yeast cells meet, karyogamy! An example of nuclear migration slowly resolved. Nucleus 4: 182–188. 23715006 10.4161/nucl.25021 PMC3720748

[bib9] Hemsley A. Arnheim N. Toney M. D. Cortopassi G. Galas D. J., 1989 A simple method for site-directed mutagenesis using the polymerase chain reaction. Nucleic Acids Res. 17: 6545–6551. 2674899 10.1093/nar/17.16.6545 PMC318348

[bib10] Hu J. Shibata Y. Zhu P.-P. Voss C. Rismanchi N., 2009 A class of dynamin-like GTPases involved in the generation of the tubular ER network. Cell 138: 549–561. 19665976 10.1016/j.cell.2009.05.025 PMC2746359

[bib11] Jaspersen S. L. Winey M., 2004 The budding yeast spindle pole body: structure, duplication, and function. Annu. Rev. Cell Dev. Biol. 20: 1–28. 15473833 10.1146/annurev.cellbio.20.022003.114106

[bib12] Kurihara L. J. Beh C. T. Latterich M. Schekman R. Rose M. D., 1994 Nuclear congression and membrane fusion: two distinct events in the yeast karyogamy pathway. J. Cell Biol. 126: 911–923. 8051211 10.1083/jcb.126.4.911 PMC2120128

[bib13] Melloy P. Shen S. White E. McIntosh J. R. Rose M. D., 2007 Nuclear fusion during yeast mating occurs by a three-step pathway. J. Cell Biol. 179: 659–670. 18025302 10.1083/jcb.200706151 PMC2080914

[bib14] Melloy P. Shen S. White E. Rose M. D., 2009 Distinct roles for key karyogamy proteins during yeast nuclear fusion. Mol. Biol. Cell 20: 3773–3782. 19570912 10.1091/mbc.E09-02-0163 PMC2735476

[bib15] Merlini L. Dudin O. Martin S. G., 2013 Mate and fuse: how yeast cells do it. Open Biol. 3: 130008. 23466674 10.1098/rsob.130008 PMC3718343

[bib16] Ng D. T. Walter P., 1996 ER membrane protein complex required for nuclear fusion. J. Cell Biol. 132: 499–509. 8647883 10.1083/jcb.132.4.499 PMC2199862

[bib17] Ning J., Otto T. D. Pfander C. Schwach F. Brochet M., , 2013 Comparative genomics in *Chlamydomonas* and *Plasmodium* identifies an ancient nuclear envelope protein family essential for sexual reproduction in protists, fungi, plants, and vertebrates. Genes Dev. 27: 1198–1215. 23699412 10.1101/gad.212746.112 PMC3672651

[bib18] Nishikawa S. Endo T., 1997 The yeast JEM1p is a DnaJ-like protein of the endoplasmic reticulum membrane required for nuclear fusion. J. Biol. Chem. 272: 12889–12892. 9148890 10.1074/jbc.272.20.12889

[bib19] Nishikawa S.-I. Terazawa Y. Nakayama T. Hirata A. Makio T., 2003 Nep98p is a component of the yeast spindle pole body and essential for nuclear division and fusion. J. Biol. Chem. 278: 9938–9943. 12493774 10.1074/jbc.M210934200

[bib20] Normington K. Kohno K. Kozutsumi Y. Gething M. J. Sambrook J., 1989 *S. cerevisiae* encodes an essential protein homologous in sequence and function to mammalian BiP. Cell 57: 1223–1236. 2661019 10.1016/0092-8674(89)90059-7

[bib21] Oldenburg K. R. Vo K. T. Michaelis S. Paddon C., 1997 Recombination-mediated PCR-directed plasmid construction *in vivo* in yeast. Nucleic Acids Res. 25: 451–452. 9016579 10.1093/nar/25.2.451 PMC146432

[bib22] Panzner S. Dreier L. Hartmann E. Kostka S. Rapoport T. A., 1995 Posttranslational protein transport in yeast reconstituted with a purified complex of Sec proteins and Kar2p. Cell 81: 561–570. 7758110 10.1016/0092-8674(95)90077-2

[bib23] Petersen T. N. Brunak S. von Heijne G. Nielsen H., 2011 SignalP 4.0: discriminating signal peptides from transmembrane regions. Nat. Methods 8: 785–786. 21959131 10.1038/nmeth.1701

[bib24] Rogers J. V. Arlow T. Inkellis E. R. Koo T. S. Rose M. D., 2013 ER-associated SNAREs and Sey1p mediate nuclear fusion at two distinct steps during yeast mating. Mol. Biol. Cell 24: 3896–3908. 24152736 10.1091/mbc.E13-08-0441 PMC3861085

[bib25] Rose M. D. Misra L. M. Vogel J. P., 1989 KAR2, a karyogamy gene, is the yeast homolog of the mammalian BiP/GRP78 gene. Cell 57: 1211–1221. 2661018 10.1016/0092-8674(89)90058-5

[bib26] Sanders S. L. Whitfield K. M. Vogel J. P. Rose M. D. Schekman R. W., 1992 Sec61p and BiP directly facilitate polypeptide translocation into the ER. Cell 69: 353–365. 1568250 10.1016/0092-8674(92)90415-9

[bib27] Sengstag C., 2000 Using *SUC2–HIS4C* reporter domain to study topology of membrane proteins in *Saccharomyces cerevisiae*. Methods Enzymol. 327: 175–190. 11044982 10.1016/s0076-6879(00)27275-3

[bib28] Shen S. Tobery C. E. Rose M. D., 2009 Prm3p is a pheromone-induced peripheral nuclear envelope protein required for yeast nuclear fusion. Mol. Biol. Cell 20: 2438–2450. 19297527 10.1091/mbc.E08-10-0987 PMC2675623

[bib29] Simons J. F. Ferro-Novick S. Rose M. D. Helenius A., 1995 BiP/Kar2p serves as a molecular chaperone during carboxypeptidase Y folding in yeast. J. Cell Biol. 130: 41–50. 7790376 10.1083/jcb.130.1.41 PMC2120506

[bib30] Tange Y. Horio T. Shimanuki M. Ding D. Q. Hiraoka Y., 1998 A novel fission yeast gene, *tht1*^+^, is required for the fusion of nuclear envelopes during karyogamy. J. Cell Biol. 140: 247–258. 9442101 10.1083/jcb.140.2.247 PMC2132580

[bib31] Vogel J. P. Misra L. M. Rose M. D., 1990 Loss of BiP/GRP78 function blocks translocation of secretory proteins in yeast. J. Cell Biol. 110: 1885–1895. 2190988 10.1083/jcb.110.6.1885 PMC2116122

[bib32] Witkin K. L. Friederichs J. M. Cohen-Fix O. Jaspersen S. L., 2010 Changes in the nuclear envelope environment affect spindle pole body duplication in *Saccharomyces cerevisiae*. Genetics 186: 867–883. 20713690 10.1534/genetics.110.119149 PMC2975299

[bib33] Ydenberg C. A. Rose M. D., 2008 Yeast mating: a model system for studying cell and nuclear fusion. Methods Mol. Biol. 475: 3–20. 18979235 10.1007/978-1-59745-250-2_1

[bib34] Young B. P. Craven R. A. Reid P. J. Willer M. Stirling C. J., 2001 Sec63p and Kar2p are required for the translocation of SRP-dependent precursors into the yeast endoplasmic reticulum *in vivo*. EMBO J. 20: 262–271. 11226176 10.1093/emboj/20.1.262 PMC140194

