# Supplementary material for: Kar5p Is Required for Multiple Functions in Both Inner and Outer Nuclear Envelope Fusion in Saccharomyces cerevisiae
Source: G3 (Bethesda). 2014 Dec 2;5(1):111–21. doi: 10.1534/g3.114.015800 (PMC4291462; doi:10.1534/g3.114.015800)
Supplement: Supporting Information [file supp_5_1_111__index.html]

Kar5p Is Required for Multiple Functions in Both Inner and Outer Nuclear Envelope Fusion in Saccharomyces cerevisiae — Supporting Information 

# Kar5p Is Required for Multiple Functions in Both Inner and Outer Nuclear Envelope Fusion in *Saccharomyces cerevisiae*

## Supporting Information for Rogers and Rose, 2015

**Files in this Data Supplement:**

- Supporting Information - Figures S1-S6 and Tables S1-S2 (PDF, 1 MB)
- Figure S1 - Quantitative comparisons of GFP-Prm3 enrichment in *kar5* mutants. (PDF, 254 KB)
- Figure S2 - Kar5-TM3-GFP is largely functional and faces outside of the ER lumen. (PDF, 177 KB)
- Figure S3 - Representative GFP images of *kar5* mutants from Figure 3. (PDF, 507 KB)
- Figure S4 - Comparison of total Kar5-TM3-GFP protein in various *kar5* mutants by western blot. (PDF, 183 KB)
- Figure S5 - Quantitative comparisons of *kar5* mutants. (PDF, 219 KB)
- Figure S6 - Complete set of unambiguous *kar5-C68A* zygote membrane bridges. (PDF, 774 KB)
- Table S1 - Strains and plasmids used in this study. (PDF, 106 KB)
- Table S2 - Raw p-values associated with Table 1 (two-sided t-test). (.xlsx, 56 KB)
