# Supplementary material for: Kar5p Is Required for Multiple Functions in Both Inner and Outer Nuclear Envelope Fusion in Saccharomyces cerevisiae
Source: G3 (Bethesda). 2014 Dec 2;5(1):111–21. doi: 10.1534/g3.114.015800 (PMC4291462; doi:10.1534/g3.114.015800)
Supplement: Supporting Information [file supp_g3.114.015800_015800SI.pdf]

G3 · Genes | Genomes | Genetics

Kar5p is required for multiple functions in both inner and outer nuclear envelope fusion in *Saccharomyces cerevisiae*

Jason V. Rogers* and Mark D. Rose*

Affiliations:

* Department of Molecular Biology, Princeton University, Princeton, NJ 08544-1014

Corresponding Author:

Mark D. Rose

Lewis Thomas Lab, Room 319, Washington Road, Princeton, NJ, 08540

Phone: (609) 258-2804

E-mail: mdrose@princeton.edu

DOI: 10.1534/g3.114.015800

Fraction GFP-Prm3 at SPB vs. Total Cellular GFP-Prm3

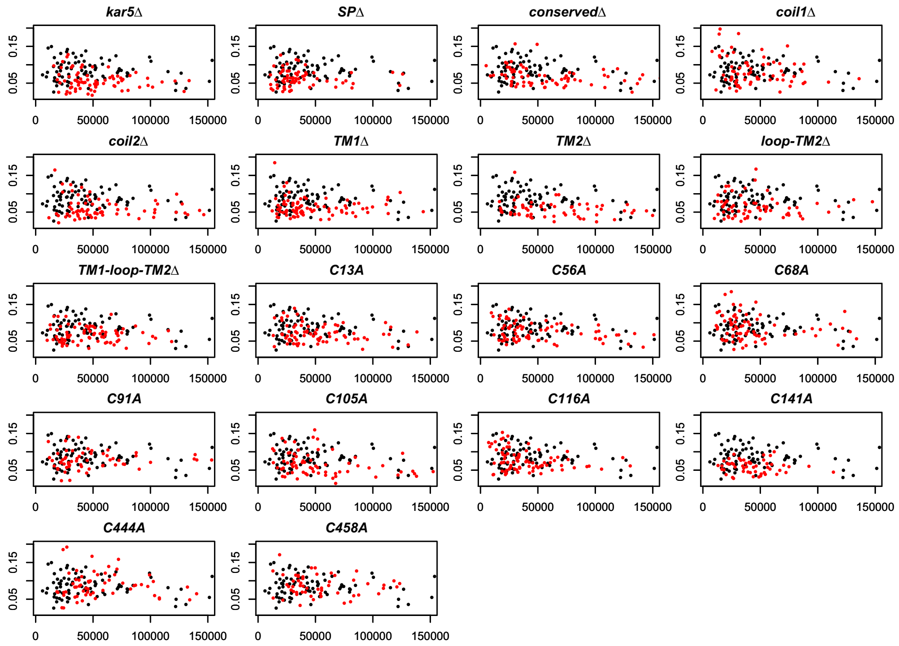

Figure S1 Quantitative comparisons of GFP-Prm3 enrichment in *kar5* mutants. Each panel shows fraction GFP-Prm3 at the SPB (y-axis) vs. total cellular GFP (x-axis). Each point is a cell; black points are *KAR5*+ (constant in each panel), and red points are the *kar5* mutant listed in the title for each panel. Data is identical to that summarized in Figure 2B. A few points lying beyond the axes are not shown for clarity.

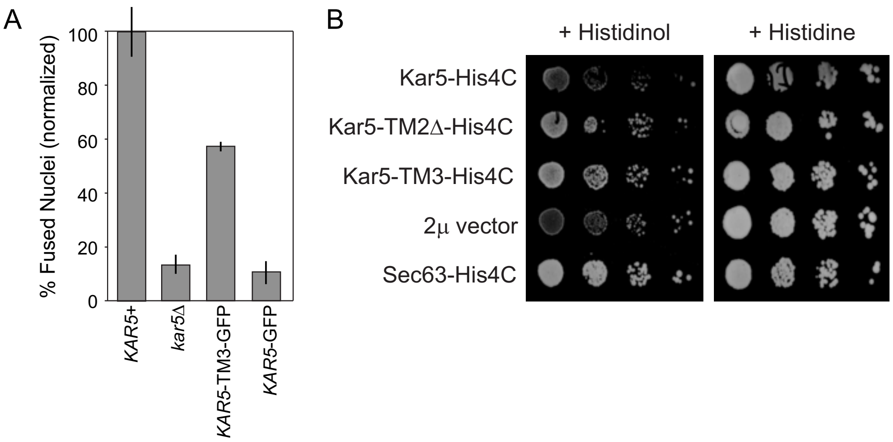

Figure S2 Kar5-TM3-GFP is largely functional and faces outside of the ER lumen. (A) Nuclear fusion assay as in Figure 1D. Average of multiple independent experiments are shown (at least three trials for each). Error bars show ±SEM. (B) His4C membrane topology assay. *his4-* cells (MY7261) containing the indicated plasmids were grown to saturation then diluted to 2.0 OD_{600} and 10-fold serial dilutions were spotted on synthetic medium containing either histidinol or histidine. Histidinol solution was prepared from dissolving L-histidinol dihydrochloride (Sigma #H 6647) to a 5% w/v aqueous solution (230 mM), adjusted to pH 9 with 10 M NaOH. Plates were prepared by spreading 250 μL of histidinol (50 mg/mL) or 100 μL histidine (10 mg/mL) liquid stock onto plates lacking uracil and histidine and allowing to dry for 1 hour prior to spotting the cells. Minor background growth was apparent in all strains on the histidinol plates, possibly due to trace amounts of contaminating histidine in the histidinol preparation (described in Sengstag 2000). All plasmids were 2μ and His4C fusions were expressed under the *ADH1* promoter.

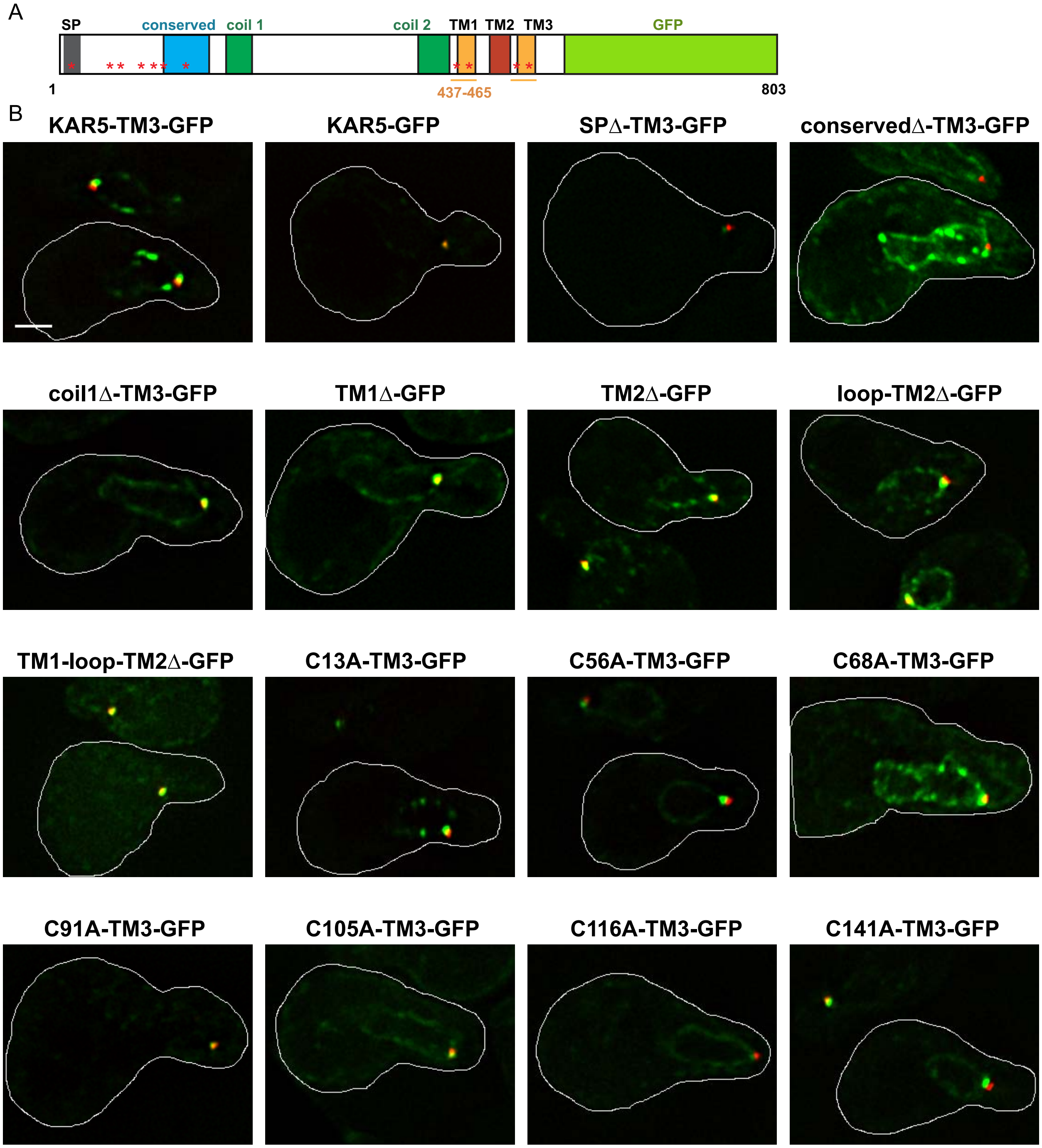

Figure S3 Representative GFP images of *kar5* mutants from Figure 3. (A) Schematic of Kar5-TM3-GFP as in Figure 3A. (B) Representative images showing a merge of GFP, Spc42-mCherry, and a cell outline. As in Figure 3, strains used are MS8020 plus the indicated CEN plasmid. All images are the same size and scaled to the same brightness/contrast. Scale bar, 2 μm.

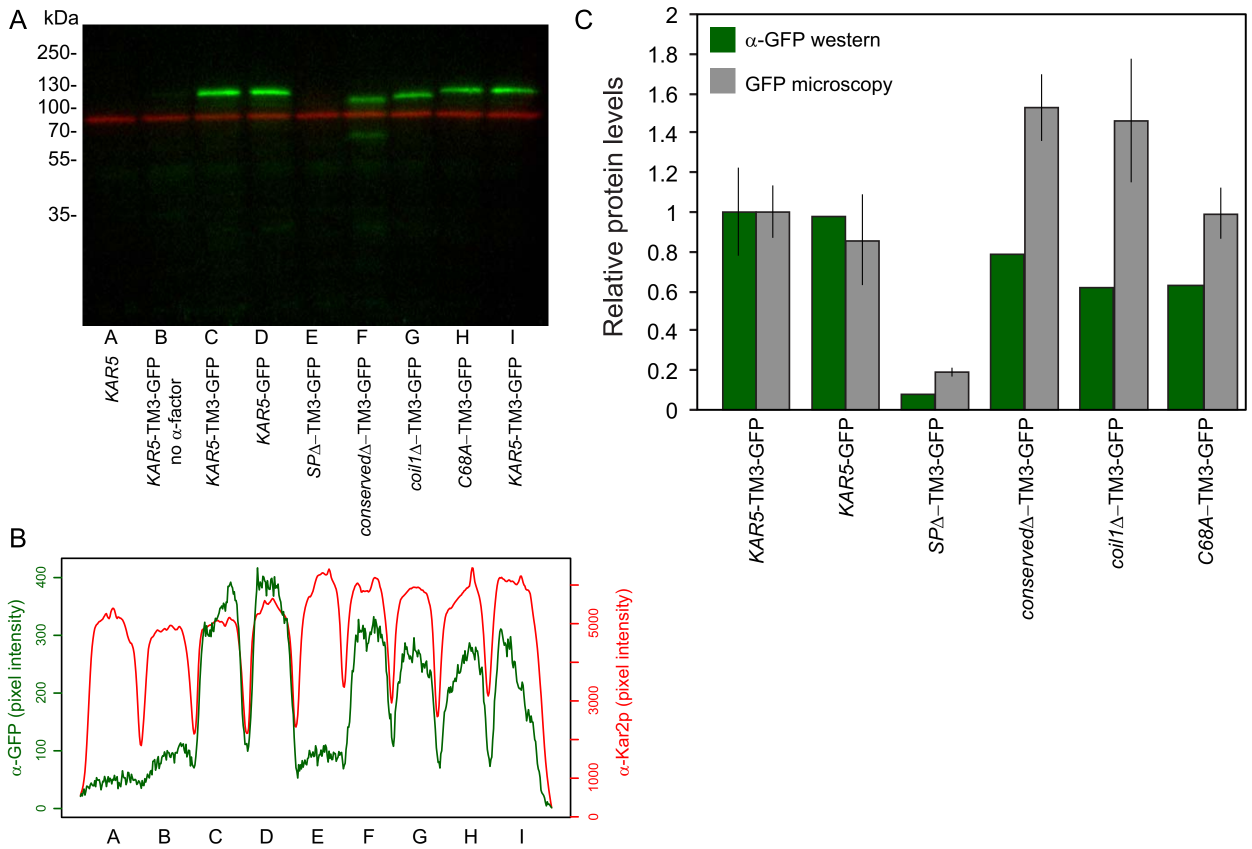

Figure S4 Comparison of total Kar5-TM3-GFP protein in various *kar5* mutants by western blot. (A) α-GFP (green; Clontech catalog # 632381) and α-Kar2p (red, custom antibody) western blot for the indicated Kar5p-GFP proteins (same strains as in Figure 3). Protein was extracted using a standard TCA precipitation and electrophoresed on a standard 10% SDS-PAGE gel under denaturing and reducing conditions. The image shown is a merge of two separate exposures for different antibodies of the same nitrocellulose membrane. Lanes C and I are technical replicates of the same protein sample. (B). Profile plot of the image shown in A for a rectangle containing the region between ~55-130 kDa. (C) Protein quantification from B, normalized to lane C (Kar5-TM3-GFP), compared to total cellular GFP data from Figure 3D.

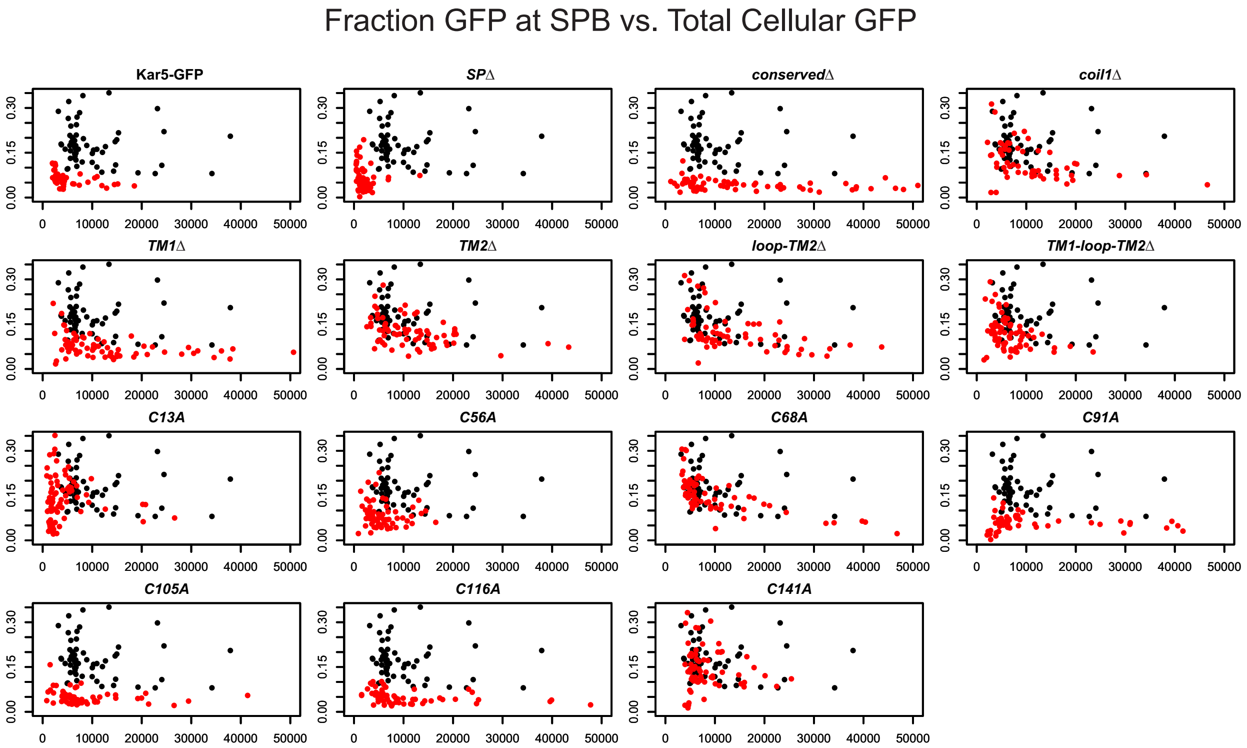

Figure S5 Quantitative comparisons of *kar5* mutants. Each panel shows fraction GFP at the SPB (y-axis) vs. total cellular GFP (x-axis). Each point is a cell; black points are Kar5-TM3-GFP (constant in each panel), and red points are the mutant listed in the title for each panel. Data is identical to that shown in Figures 3D and 3E. A few points lying beyond the axes are not shown for clarity. From these plots, the expression and SPB enrichment ability relative to Kar5-TM3-GFP is easily visualized for each mutant. For example, the *C13A* mutant has less total GFP on average, but the fraction GFP at the SPB is similar. Conversely, the *conserved*Δ mutant has normal GFP expression levels, but the fraction at the SPB is consistently low.

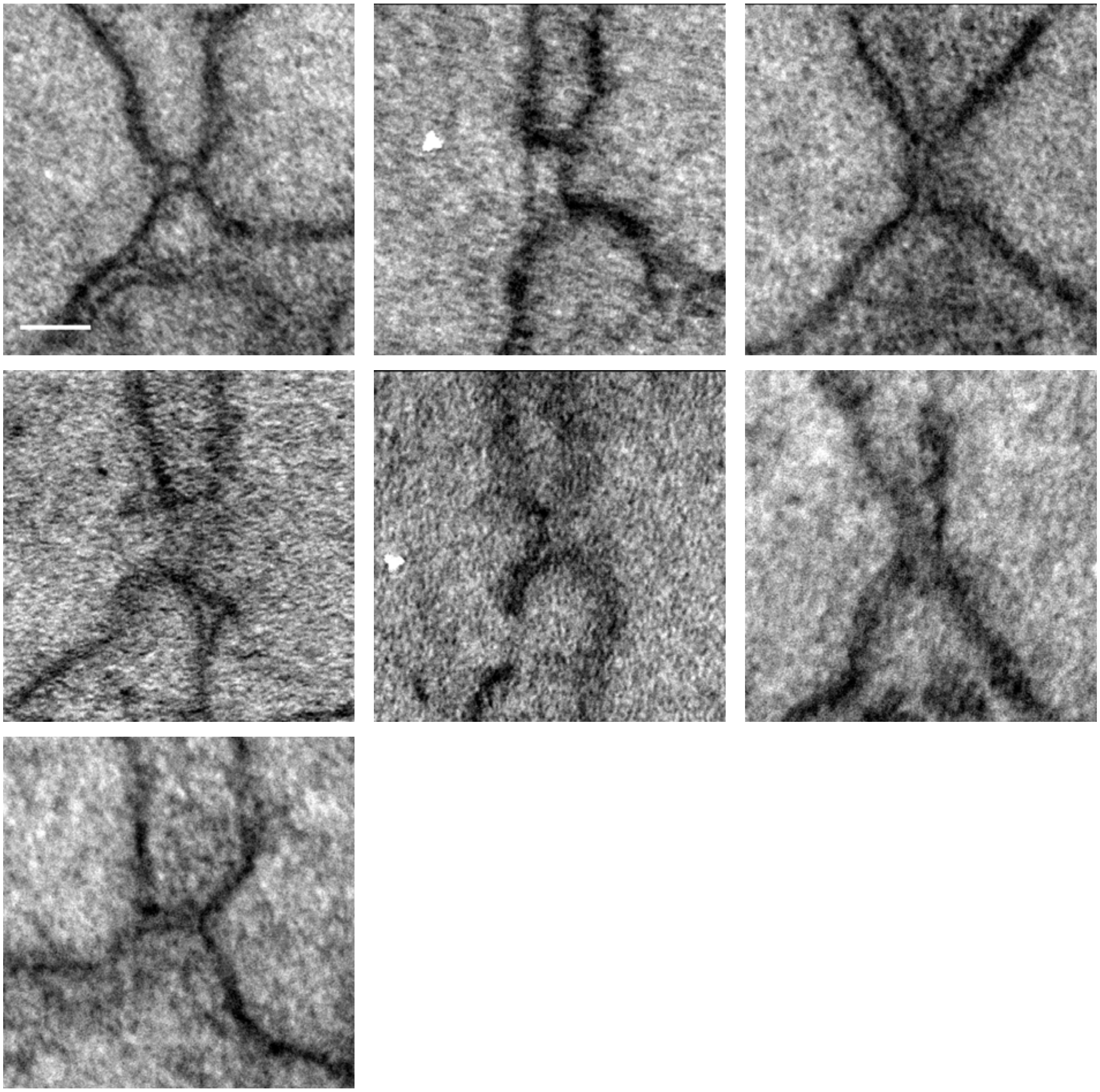

Figure S6 Complete set of unambiguous *kar5-C68A* zygote membrane bridges. Images shown are from the same experiment discussed in Figure 6. Note that the first six bridges appear wide and expanded, whereas the final bridge is thin and long. Each image is a 500 x 500 nm area; scale bar is 100 nm.

Table S1   Strains and plasmids used in this study

Strain	Genotype	Source	Notes
MS7590	*MAT**a** prm3Δ::HIS3 ura3-52 leu2-3,112 trp1Δ1 his3Δ200*	Rose Laboratory	
MS7591	*MATα prm3Δ::HIS3 ura3-52 leu2-3,112 ade2-101 his3Δ200*	Rose Laboratory	
MS7670	*MAT**a** kar5Δ::HIS3 ura3-52 leu2-3,112 his3Δ200 trp1Δ1*	Rose Laboratory	
MS7673	*MATα kar5Δ::HIS3 ura3-52 leu2-3,112 his3Δ200 trp1Δ1*	Rose Laboratory	
MS7729	*MAT**a** ura3-52 leu2-3,112 his3Δ200 trp1Δ1 ade2-101 SPC42::mRFP-KanMX*	Rose Laboratory	
MS7884	*MAT**a** prm3Δ::HIS3 ura3-52 leu2-3,112 his3Δ200 trp1Δ1 ade2-101 SPC42::mRFP-KanMX*	Rose Laboratory	
MS8020	*MAT**a** kar5Δ::HIS3 ura3-52 leu2-3,112 his3Δ200 trp1Δ1 SPC42::mCherry-KanMX*	This study	
MS8041	*MAT**a** kar2-1 ura3-52 leu2-3,112 trp1Δ1 SPC42::mCherry-KanMX*	This study	
MS8087	*MAT**a** kar8Δ::NatMX kar5Δ::HIS3 ura3-52 leu2-3,112 his3Δ200 trp1Δ1 SPC42::mCherry-KanMX*	This study	
MS8389	*MAT**a** kar5Δ::HIS3 ura3-52 leu2-3,112 his3Δ200 trp1Δ1 SPC42::mCherry-KanMX prm3Δ::NatMX [GFP-PRM3 LEU2 CEN4]*	This study	
MY7261	*MAT**a** his4- HOL1-1 ura3-52*	Rose Laboratory	
MY14348	*MAT**a** pom152Δ::HygMX ade2-1 his3-11,15 leu2-3,112 trp1Δ1 ura3Δ1 ade3Δ100*	This study	
MY14349	*MATα pom152Δ::HygMX ade2-1 his3-11,15 leu2-3,112 trp1Δ1 ura3Δ1*	This study	
MY14355	*MAT**a** pom152Δ::HygMX mps3Δ::NatMX ade2-1 his3-11,15 leu2-3,112 trp1Δ1 ura3Δ1*	This study	
MY14357	*MATα pom152Δ::HygMX mps3Δ::NatMX ade2-1 his3-11,15 leu2-3,112 trp1Δ1 ura3Δ1*	This study	parent strain for all *pom152Δ mps3Δ* strains from Sue Jaspersen
MY14635	*MAT**a** pom152Δ::HygMX ade2-1 his3-11,15 leu2-3,112 trp1Δ1 ura3Δ1 ade3Δ100 SPC42::mCherry-KanMX*	This study	
MY14637	*MAT**a** pom152Δ::HygMX mps3Δ::NatMX ade2-1 his3-11,15 leu2-3,112 trp1Δ1 ura3Δ1 SPC42::mCherry-KanMX*	This study	
MY14667	*MAT**a** [GFP11-mCherry-PUS1-LEU2] trp- his- ura-*	Jaspersen Laboratory	
MY14668	*MAT**a** [GFP11-mCherry-SCS2TM-LEU2] lys- trp- his- ura-*	Jaspersen Laboratory	
**Plasmid**	**Relevant markers**	**Source**	
pMR1868	*URA3 CEN6 ARS4 amp-r*	Rose Laboratory	
pMR4518	*KAR5 URA3 CEN6 ARS4 amp-r*	Rose Laboratory	
pMR6603	*KAR5(C13A) URA3 CEN6 ARS4 amp-r*	This study	
pMR6381	*KAR5(C56A) URA3 CEN6 ARS4 amp-r*	This study	
pMR6382	*KAR5(C68A) URA3 CEN6 ARS4 amp-r*	This study	
pMR6383	*KAR5(C91A) URA3 CEN6 ARS4 amp-r*	This study	
pMR6604	*KAR5(C105A) URA3 CEN6 ARS4 amp-r*	This study	
pMR6605	*KAR5(C116A) URA3 CEN6 ARS4 amp-r*	This study	
pMR6606	*KAR5(C141A) URA3 CEN6 ARS4 amp-r*	This study	
pMR6384	*KAR5(C444A) URA3 CEN6 ARS4 amp-r*	This study	
pMR6607	*KAR5(C458A) URA3 CEN6 ARS4 amp-r*	This study	
pMR6608	*KAR5(Δ4-23) URA3 CEN6 ARS4 amp-r (SPΔ)*	This study	
pMR6385	*KAR5(Δ186-215) URA3 CEN6 ARS4 amp-r (coil1Δ)*	This study	
pMR6386	*KAR5(Δ401-436) URA3 CEN6 ARS4 amp-r (coil2Δ)*	This study	
pMR6609	*KAR5(Δ445-465) URA3 CEN6 ARS4 amp-r (TM1Δ)*	This study	

pMR6387	*KAR5(Δ481-504) URA3 CEN6 ARS4 amp-r (TM2Δ)*	This study
pMR6751	*KAR5(Δ466-504) URA3 CEN6 ARS4 amp-r (loop-TM2Δ)*	This study
pMR6610	*KAR5(Δ445-504) URA3 CEN6 ARS4 amp-r (TM1-loop-TM2Δ)*	This study
pMR6611	*KAR5(Δ116-167) URA3 CEN6 ARS4 amp-r (conserved regionΔ)*	This study
pMR6366	*KAR5:GFP URA3 CEN6 ARS4 amp-r*	This study
pMR6364	*KAR5:TM3:GFP URA3 CEN6 ARS4 amp-r*	This study
pMR6612	*KAR5(C13A):TM3:GFP URA3 CEN6 ARS4 amp-r*	This study
pMR6749	*KAR5(C56A):TM3:GFP URA3 CEN6 ARS4 amp-r*	This study
pMR6750	*KAR5(C68A):TM3:GFP URA3 CEN6 ARS4 amp-r*	This study
pMR6615	*KAR5(C91A):TM3:GFP URA3 CEN6 ARS4 amp-r*	This study
pMR6616	*KAR5(C105A):TM3:GFP URA3 CEN6 ARS4 amp-r*	This study
pMR6617	*KAR5(C116A):TM3:GFP URA3 CEN6 ARS4 amp-r*	This study
pMR6618	*KAR5(C141A):TM3:GFP URA3 CEN6 ARS4 amp-r*	This study
pMR6619	*KAR5(Δ4-23):TM3:GFP URA3 CEN6 ARS4 amp-r (SPΔ)*	This study
pMR6620	*KAR5(Δ186-215):TM3:GFP URA3 CEN6 ARS4 amp-r (coil1Δ)*	This study
pMR6622	*KAR5(Δ116-167):TM3:GFP URA3 CEN6 ARS4 amp-r (conserved regionΔ)*	This study
pMR6627	*KAR5(Δ445-465):GFP URA3 CEN6 ARS4 amp-r (TM1Δ)*	This study
pMR6748	*KAR5(Δ481-504):GFP URA3 CEN6 ARS4 amp-r (TM2Δ)*	This study
pMR6629	*KAR5(Δ466-504):GFP URA3 CEN6 ARS4 amp-r (loop-TM2Δ)*	This study
pMR6744	*KAR5(Δ445-504):GFP URA3 CEN6 ARS4 amp-r (TM1-loop-TM2Δ)*	This study
pMR6082	*pADH1-KAR5:HIS4C URA3 2μ amp-r*	This study
pMR6081	*pADH1-KAR5:TM3:HIS4C URA3 2μ amp-r*	This study
pMR6083	*pADH1-KAR5(TM2Δ):HIS4C URA3 2μ amp-r*	This study
pMR6085	*pADH1-SEC63:HIS4C URA3 2μ amp-r*	This study
pMR1872	*URA3 2μ amp-r*	Rose Laboratory
pMR6371	*pADH1-KAR5:TM3:GFP URA3 CEN6 ARS4 amp-r*	This study
pMR6433	*pADH1-mCherry:PRM3 LEU2 CEN6 ARS4 amp-r*	This study
pMR6907	*pADH1-GFP1-10 URA3 CEN6 ARS4 amp-r*	This study
pMR6945	*pSEY1-SEY1-GFP1-10 URA3 CEN6 ARS4 amp-r*	This study
pMR6932	*pKAR5-KAR5:TM3:GFP1-10 URA3 CEN6 ARS4 amp-r*	This study

Table S2 Raw p-values associated with Table 1 (two-sided t-test)

Available for download as an Excel file at http://www.g3journal.org/lookup/suppl/doi:10.1534/g3.114.015800/-/DC1
