# Supplementary material for: Kar5p Is Required for Multiple Functions in Both Inner and Outer Nuclear Envelope Fusion in Saccharomyces cerevisiae
Source: G3 (Bethesda). 2014 Dec 2;5(1):111–21. doi: 10.1534/g3.114.015800 (PMC4291462; doi:10.1534/g3.114.015800)
Supplement: Supporting Information [file supp_g3.114.015800_FigureS1.pdf]

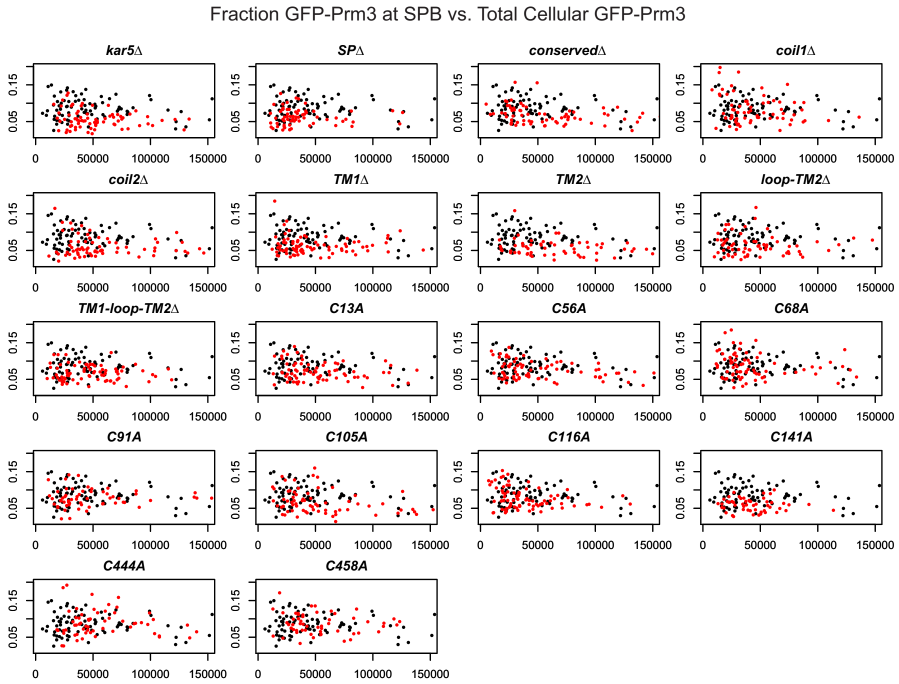

Figure S1 Quantitative comparisons of GFP-Prm3 enrichment in *kar5* mutants. Each panel shows fraction GFP-Prm3 at the SPB (y-axis) vs. total cellular GFP (x-axis). Each point is a cell; black points are *KAR5*+ (constant in each panel), and red points are the *kar5* mutant listed in the title for each panel. Data is identical to that summarized in Figure 2B. A few points lying beyond the axes are not shown for clarity.
