# Supplementary material for: Kar5p Is Required for Multiple Functions in Both Inner and Outer Nuclear Envelope Fusion in Saccharomyces cerevisiae
Source: G3 (Bethesda). 2014 Dec 2;5(1):111–21. doi: 10.1534/g3.114.015800 (PMC4291462; doi:10.1534/g3.114.015800)
Supplement: Supporting Information [file supp_g3.114.015800_FigureS2.pdf]

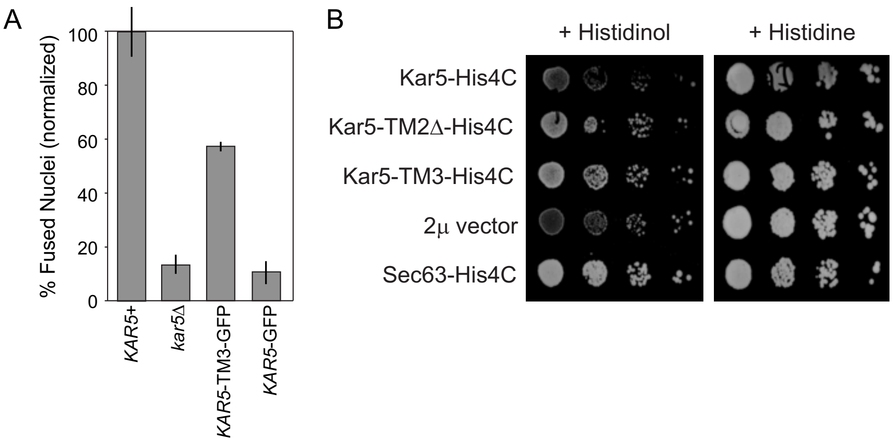

Figure S2 Kar5-TM3-GFP is largely functional and faces outside of the ER lumen. (A) Nuclear fusion assay as in Figure 1D. Average of multiple independent experiments are shown (at least three trials for each). Error bars show ±SEM. (B) His4C membrane topology assay. *his4*- cells (MY7261) containing the indicated plasmids were grown to saturation then diluted to 2.0 OD_{600} and 10-fold serial dilutions were spotted on synthetic medium containing either histidinol or histidine. Histidinol solution was prepared from dissolving L-histidinol dihydrochloride (Sigma #H 6647) to a 5% w/v aqueous solution (230 mM), adjusted to pH 9 with 10 M NaOH. Plates were prepared by spreading 250 µL of histidinol (50 mg/mL) or 100 µL histidine (10 mg/mL) liquid stock onto plates lacking uracil and histidine and allowing to dry for 1 hour prior to spotting the cells. Minor background growth was apparent in all strains on the histidinol plates, possibly due to trace amounts of contaminating histidine in the histidinol preparation (described in Sengstag 2000). All plasmids were 2µ and His4C fusions were expressed under the *ADH1* promoter.
