# Supplementary material for: Kar5p Is Required for Multiple Functions in Both Inner and Outer Nuclear Envelope Fusion in Saccharomyces cerevisiae
Source: G3 (Bethesda). 2014 Dec 2;5(1):111–21. doi: 10.1534/g3.114.015800 (PMC4291462; doi:10.1534/g3.114.015800)
Supplement: Supporting Information [file supp_g3.114.015800_FigureS3.pdf]

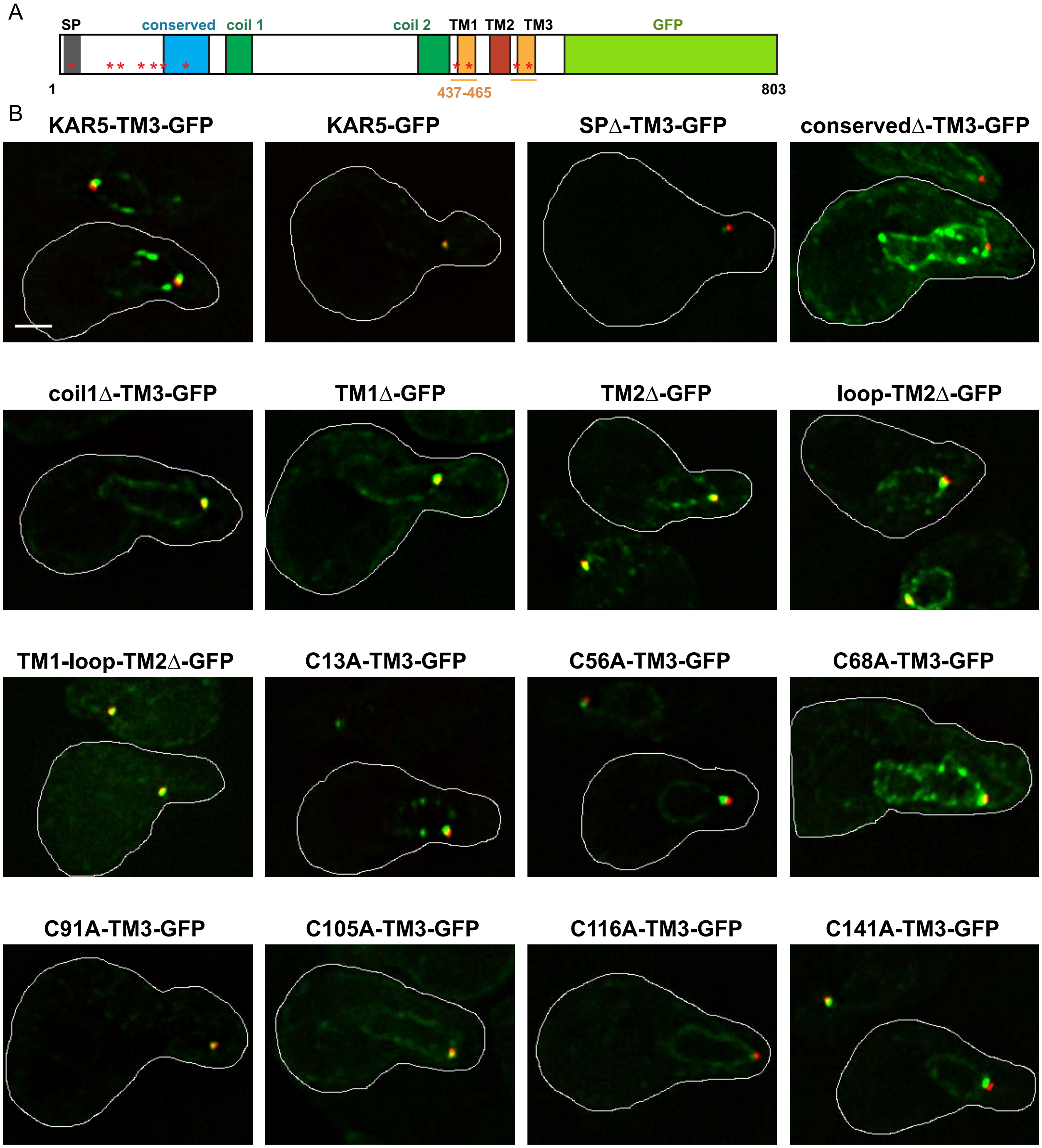

Figure S3 Representative GFP images of *kar5* mutants from Figure 3. (A) Schematic of Kar5-TM3-GFP as in Figure 3A. (B) Representative images showing a merge of GFP, Spc42-mCherry, and a cell outline. As in Figure 3, strains used are MS8020 plus the indicated CEN plasmid. All images are the same size and scaled to the same brightness/contrast. Scale bar, 2 μm.
