# Supplementary material for: Kar5p Is Required for Multiple Functions in Both Inner and Outer Nuclear Envelope Fusion in Saccharomyces cerevisiae
Source: G3 (Bethesda). 2014 Dec 2;5(1):111–21. doi: 10.1534/g3.114.015800 (PMC4291462; doi:10.1534/g3.114.015800)
Supplement: Supporting Information [file supp_g3.114.015800_FigureS4.pdf]

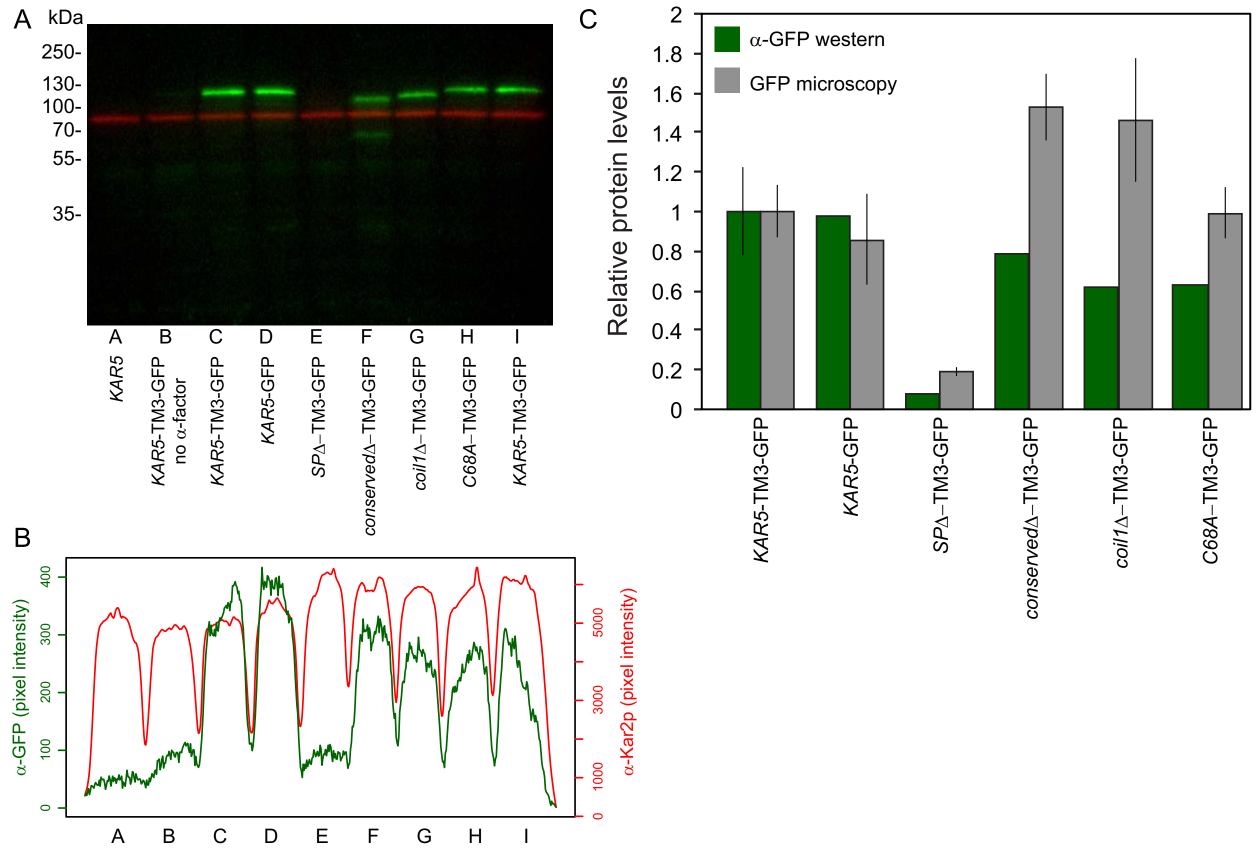

Figure S4 Comparison of total Kar5-TM3-GFP protein in various *kar5* mutants by western blot. (A) α-GFP (green; Clontech catalog # 632381) and α-Kar2p (red, custom antibody) western blot for the indicated Kar5p-GFP proteins (same strains as in Figure 3). Protein was extracted using a standard TCA precipitation and electrophoresed on a standard 10% SDS-PAGE gel under denaturing and reducing conditions. The image shown is a merge of two separate exposures for different antibodies of the same nitrocellulose membrane. Lanes C and I are technical replicates of the same protein sample. (B). Profile plot of the image shown in A for a rectangle containing the region between ~55-130 kDa. (C) Protein quantification from B, normalized to lane C (Kar5-TM3-GFP), compared to total cellular GFP data from Figure 3D.
