# Supplementary material for: Kar5p Is Required for Multiple Functions in Both Inner and Outer Nuclear Envelope Fusion in Saccharomyces cerevisiae
Source: G3 (Bethesda). 2014 Dec 2;5(1):111–21. doi: 10.1534/g3.114.015800 (PMC4291462; doi:10.1534/g3.114.015800)
Supplement: Supporting Information [file supp_g3.114.015800_FigureS5.pdf]

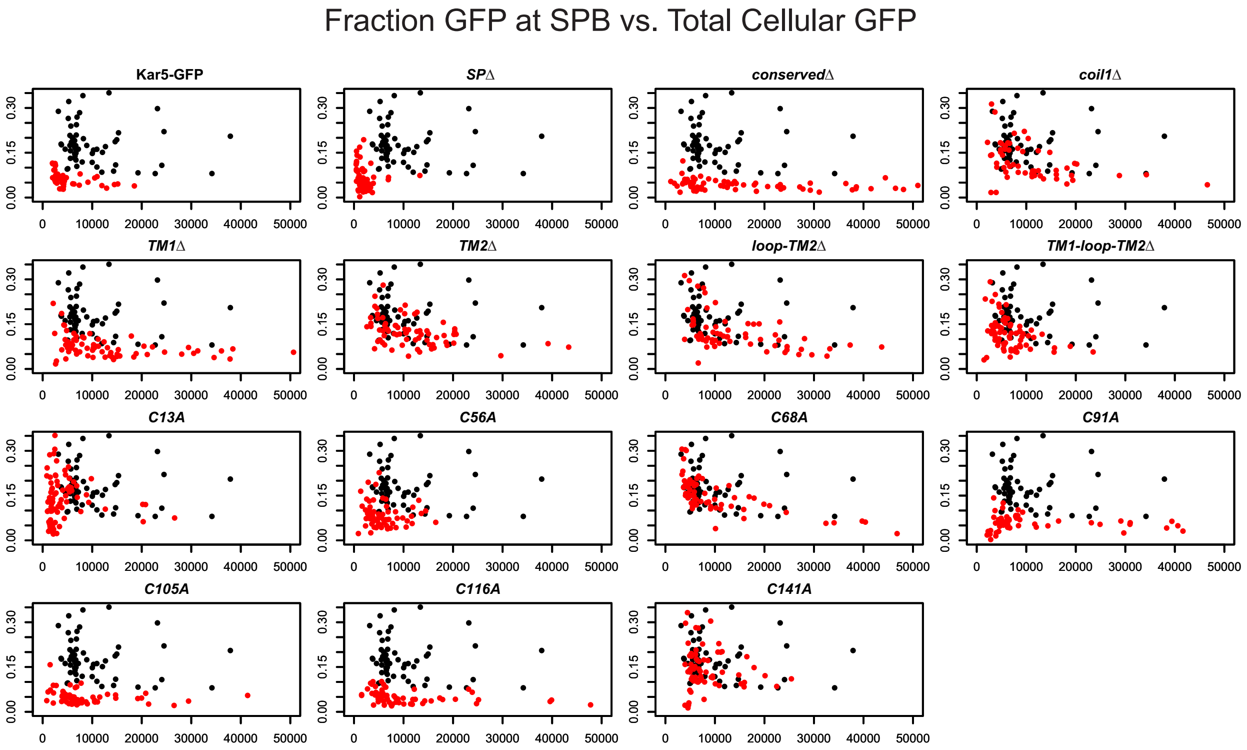

Figure S5 Quantitative comparisons of *kar5* mutants. Each panel shows fraction GFP at the SPB (y-axis) vs. total cellular GFP (x-axis). Each point is a cell; black points are Kar5-TM3-GFP (constant in each panel), and red points are the mutant listed in the title for each panel. Data is identical to that shown in Figures 3D and 3E. A few points lying beyond the axes are not shown for clarity. From these plots, the expression and SPB enrichment ability relative to Kar5-TM3-GFP is easily visualized for each mutant. For example, the *C13A* mutant has less total GFP on average, but the fraction GFP at the SPB is similar. Conversely, the *conserved*Δ mutant has normal GFP expression levels, but the fraction at the SPB is consistently low.
