# Supplementary material for: Kar5p Is Required for Multiple Functions in Both Inner and Outer Nuclear Envelope Fusion in Saccharomyces cerevisiae
Source: G3 (Bethesda). 2014 Dec 2;5(1):111–21. doi: 10.1534/g3.114.015800 (PMC4291462; doi:10.1534/g3.114.015800)
Supplement: Supporting Information [file supp_g3.114.015800_FigureS6.pdf]

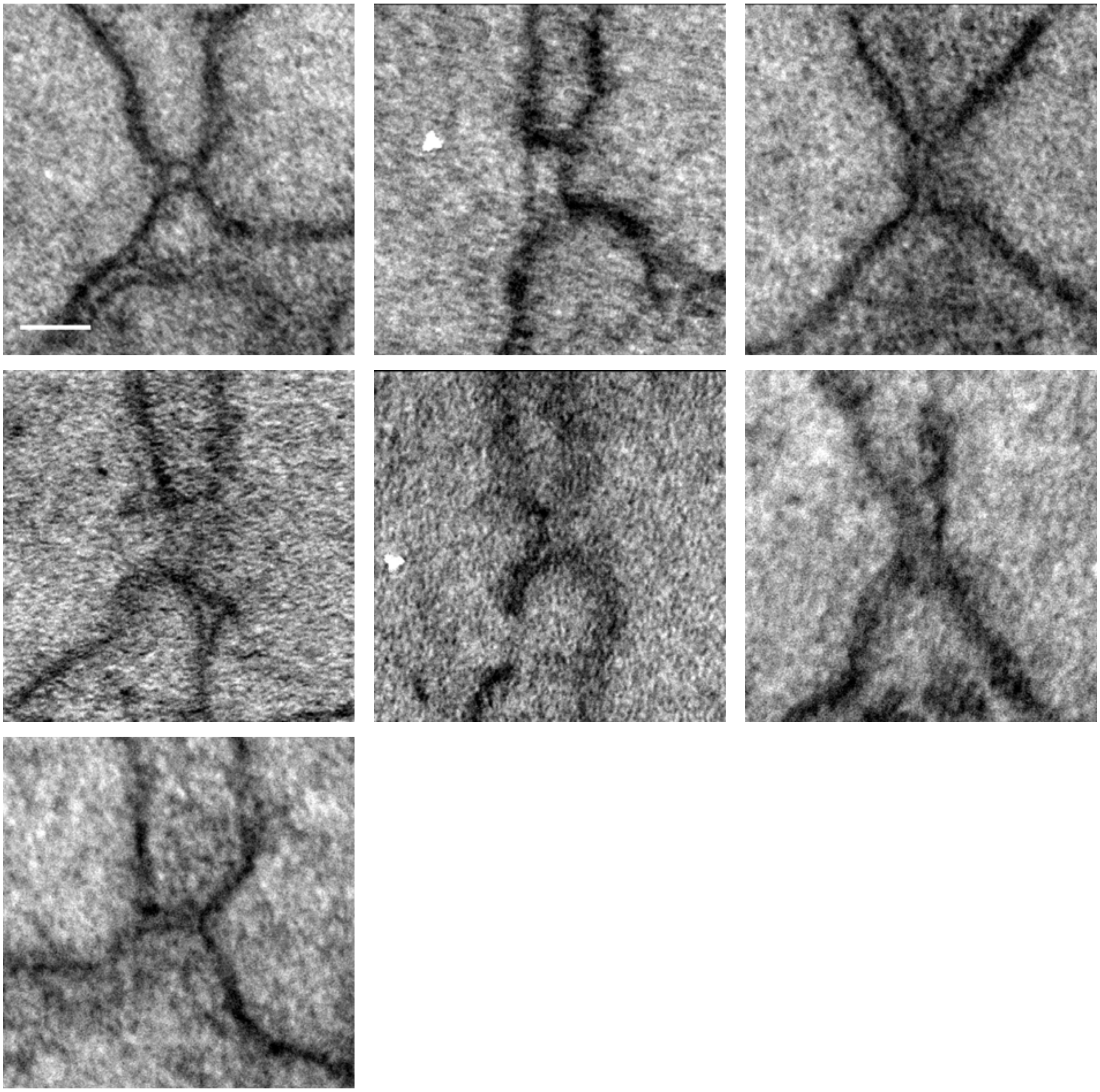

Figure S6 Complete set of unambiguous *kar5-C68A* zygote membrane bridges. Images shown are from the same experiment discussed in Figure 6. Note that the first six bridges appear wide and expanded, whereas the final bridge is thin and long. Each image is a 500 x 500 nm area; scale bar is 100 nm.
