# Supplementary material for: Kar5p Is Required for Multiple Functions in Both Inner and Outer Nuclear Envelope Fusion in Saccharomyces cerevisiae
Source: G3 (Bethesda). 2014 Dec 2;5(1):111–21. doi: 10.1534/g3.114.015800 (PMC4291462; doi:10.1534/g3.114.015800)
Supplement: Supporting Information [file supp_g3.114.015800_TableS1.pdf]

**Table S1 Strains and plasmids used in this study**

| Strain | Genotype | Source | Notes |
|---|---|---|---|
| MS7590 | *MAT**a** prm3Δ::HIS3 ura3-52 leu2-3,112 trp1Δ1 his3Δ200* | Rose Laboratory | |
| MS7591 | *MATα prm3Δ::HIS3 ura3-52 leu2-3,112 ade2-101 his3Δ200* | Rose Laboratory | |
| MS7670 | *MAT**a** kar5Δ::HIS3 ura3-52 leu2-3,112 his3Δ200 trp1Δ1* | Rose Laboratory | |
| MS7673 | *MATα kar5Δ::HIS3 ura3-52 leu2-3,112 his3Δ200 trp1Δ1* | Rose Laboratory | |
| MS7729 | *MAT**a** ura3-52 leu2-3,112 his3Δ200 trp1Δ1 ade2-101 SPC42::mRFP-KanMX* | Rose Laboratory | |
| MS7884 | *MAT**a** prm3Δ::HIS3 ura3-52 leu2-3,112 his3Δ200 trp1Δ1 ade2-101 SPC42::mRFP-KanMX* | Rose Laboratory | |
| MS8020 | *MAT**a** kar5Δ::HIS3 ura3-52 leu2-3,112 his3Δ200 trp1Δ1 SPC42::mCherry-KanMX* | This study | |
| MS8041 | *MAT**a** kar2-1 ura3-52 leu2-3,112 trp1Δ1 SPC42::mCherry-KanMX* | This study | |
| MS8087 | *MAT**a** kar8Δ::NatMX kar5Δ::HIS3 ura3-52 leu2-3,112 his3Δ200 trp1Δ1 SPC42::mCherry-KanMX* | This study | |
| MS8389 | *MAT**a** kar5Δ::HIS3 ura3-52 leu2-3,112 his3Δ200 trp1Δ1 SPC42::mCherry-KanMX prm3Δ::NatMX [GFP-PRM3 LEU2 CEN4]* | This study | |
| MY7261 | *MAT**a** his4- HOL1-1 ura3-52* | Rose Laboratory | |
| MY14348 | *MAT**a** pom152Δ::HygMX ade2-1 his3-11,15 leu2-3,112 trp1Δ1 ura3Δ1 ade3Δ100* | This study | |
| MY14349 | *MATα pom152Δ::HygMX ade2-1 his3-11,15 leu2-3,112 trp1Δ1 ura3Δ1* | This study | |
| MY14355 | *MAT**a** pom152Δ::HygMX mps3Δ::NatMX ade2-1 his3-11,15 leu2-3,112 trp1Δ1 ura3Δ1* | This study | |
| MY14357 | *MATα pom152Δ::HygMX mps3Δ::NatMX ade2-1 his3-11,15 leu2-3,112 trp1Δ1 ura3Δ1* | This study | parent strain for all *pom152Δ mps3Δ* strains from Sue Jaspersen |
| MY14635 | *MAT**a** pom152Δ::HygMX ade2-1 his3-11,15 leu2-3,112 trp1Δ1 ura3Δ1 ade3Δ100 SPC42::mCherry-KanMX* | This study | |
| MY14637 | *MAT**a** pom152Δ::HygMX mps3Δ::NatMX ade2-1 his3-11,15 leu2-3,112 trp1Δ1 ura3Δ1 SPC42::mCherry-KanMX* | This study | |
| MY14667 | *MAT**a** [GFP11-mCherry-PUS1-LEU2] trp- his- ura-* | Jaspersen Laboratory | |
| MY14668 | *MAT**a** [GFP11-mCherry-SCS2TM-LEU2] lys- trp- his- ura-* | Jaspersen Laboratory | |

| Plasmid | Relevant markers | Source |
|---|---|---|
| pMR1868 | *URA3 CEN6 ARS4 amp-r* | Rose Laboratory |
| pMR4518 | *KAR5 URA3 CEN6 ARS4 amp-r* | Rose Laboratory |
| pMR6603 | *KAR5(C13A) URA3 CEN6 ARS4 amp-r* | This study |
| pMR6381 | *KAR5(C56A) URA3 CEN6 ARS4 amp-r* | This study |
| pMR6382 | *KAR5(C68A) URA3 CEN6 ARS4 amp-r* | This study |
| pMR6383 | *KAR5(C91A) URA3 CEN6 ARS4 amp-r* | This study |
| pMR6604 | *KAR5(C105A) URA3 CEN6 ARS4 amp-r* | This study |
| pMR6605 | *KAR5(C116A) URA3 CEN6 ARS4 amp-r* | This study |
| pMR6606 | *KAR5(C141A) URA3 CEN6 ARS4 amp-r* | This study |
| pMR6384 | *KAR5(C444A) URA3 CEN6 ARS4 amp-r* | This study |
| pMR6607 | *KAR5(C458A) URA3 CEN6 ARS4 amp-r* | This study |
| pMR6608 | *KAR5(Δ4-23) URA3 CEN6 ARS4 amp-r (SPΔ)* | This study |
| pMR6385 | *KAR5(Δ186-215) URA3 CEN6 ARS4 amp-r (coil1Δ)* | This study |
| pMR6386 | *KAR5(Δ401-436) URA3 CEN6 ARS4 amp-r (coil2Δ)* | This study |
| pMR6609 | *KAR5(Δ445-465) URA3 CEN6 ARS4 amp-r (TM1Δ)* | This study |

| | | |
|---|---|---|
| pMR6387 | *KAR5(Δ481-504) URA3 CEN6 ARS4 amp-r (TM2Δ)* | This study |
| pMR6751 | *KAR5(Δ466-504) URA3 CEN6 ARS4 amp-r (loop-TM2Δ)* | This study |
| pMR6610 | *KAR5(Δ445-504) URA3 CEN6 ARS4 amp-r (TM1-loop-TM2Δ)* | This study |
| pMR6611 | *KAR5(Δ116-167) URA3 CEN6 ARS4 amp-r (conserved regionΔ)* | This study |
| pMR6366 | *KAR5:GFP URA3 CEN6 ARS4 amp-r* | This study |
| pMR6364 | *KAR5:TM3:GFP URA3 CEN6 ARS4 amp-r* | This study |
| pMR6612 | *KAR5(C13A):TM3:GFP URA3 CEN6 ARS4 amp-r* | This study |
| pMR6749 | *KAR5(C56A):TM3:GFP URA3 CEN6 ARS4 amp-r* | This study |
| pMR6750 | *KAR5(C68A):TM3:GFP URA3 CEN6 ARS4 amp-r* | This study |
| pMR6615 | *KAR5(C91A):TM3:GFP URA3 CEN6 ARS4 amp-r* | This study |
| pMR6616 | *KAR5(C105A):TM3:GFP URA3 CEN6 ARS4 amp-r* | This study |
| pMR6617 | *KAR5(C116A):TM3:GFP URA3 CEN6 ARS4 amp-r* | This study |
| pMR6618 | *KAR5(C141A):TM3:GFP URA3 CEN6 ARS4 amp-r* | This study |
| pMR6619 | *KAR5(Δ4-23):TM3:GFP URA3 CEN6 ARS4 amp-r (SPΔ)* | This study |
| pMR6620 | *KAR5(Δ186-215):TM3:GFP URA3 CEN6 ARS4 amp-r (coil1Δ)* | This study |
| pMR6622 | *KAR5(Δ116-167):TM3:GFP URA3 CEN6 ARS4 amp-r (conserved regionΔ)* | This study |
| pMR6627 | *KAR5(Δ445-465):GFP URA3 CEN6 ARS4 amp-r (TM1Δ)* | This study |
| pMR6748 | *KAR5(Δ481-504):GFP URA3 CEN6 ARS4 amp-r (TM2Δ)* | This study |
| pMR6629 | *KAR5(Δ466-504):GFP URA3 CEN6 ARS4 amp-r (loop-TM2Δ)* | This study |
| pMR6744 | *KAR5(Δ445-504):GFP URA3 CEN6 ARS4 amp-r (TM1-loop-TM2Δ)* | This study |
| pMR6082 | *pADH1-KAR5:HIS4C URA3 2μ amp-r* | This study |
| pMR6081 | *pADH1-KAR5:TM3:HIS4C URA3 2μ amp-r* | This study |
| pMR6083 | *pADH1-KAR5(TM2Δ):HIS4C URA3 2μ amp-r* | This study |
| pMR6085 | *pADH1-SEC63:HIS4C URA3 2μ amp-r* | This study |
| pMR1872 | *URA3 2μ amp-r* | Rose Laboratory |
| pMR6371 | *pADH1-KAR5:TM3:GFP URA3 CEN6 ARS4 amp-r* | This study |
| pMR6433 | *pADH1-mCherry:PRM3 LEU2 CEN6 ARS4 amp-r* | This study |
| pMR6907 | *pADH1-GFP1-10 URA3 CEN6 ARS4 amp-r* | This study |
| pMR6945 | *pSEY1-SEY1-GFP1-10  URA3 CEN6 ARS4 amp-r* | This study |
| pMR6932 | *pKAR5-KAR5:TM3:GFP1-10 URA3 CEN6 ARS4 amp-r* | This study |

**Table S2   Raw p-values associated with Table 1 (two-sided t-test)**

Available for download as an Excel file at http://www.g3journal.org/lookup/suppl/doi:10.1534/g3.114.015800/-/DC1
